# Drug hypersensitivity reactions in children in clinical practice: A WAO Statement^☆^

**DOI:** 10.1016/j.waojou.2025.101087

**Published:** 2025-08-29

**Authors:** Steering Committee Authors, M. Pilar Berges-Gimeno, Emilio Alvarez-Cuesta, Marina Atanaskovic-Markovic, Marina Attanassi, Carlo Caffareli, Jean-Christoph Caubet, George du Toit, Antonieta Guzman-Melendez, Semanur Kuyucu, Ricardo Madrigal-Burgaleta, Lina Mayorga, Elizabeth Powell, Michelle Ramien, Eva Rebelo Gomes, Francesca Mori, Andrew A. White, Ignacio J. Ansotegui, Review Panel Authors, Marcelo Vivolo Aun, Annick Barbaud, Sevim Bavbek, Lorena Bernal-Rubio, Knut Brockow, Lucrecia Bustamante, Yoon-Seok Chang, Luis Felipe Chiaverini Ensina, Javier Cuesta-Herranz, Bryan N. Fernandes, Lene Heise Garvey, Pedro Giavina-Bianchi, Mona I. Kidon, Marina Labella Alvarez, Joanna S. Makowska, Susana Marinho, David Alejandro Mendoza-Hernández, Mauro Pagani, Valeria Palma Pino, Claudio A.S. Parisi, Hae-Sim Park, Jonathan Peter, Elizabeth J. Phillips, Kimberly Risma, Emilio Solano-Solares, Luciana Kase Tanno, Rocco Luigi Valluzzi, Paula Vazquez-Revuelta, Timothy J. Watts, Masao Yamaguchi

**Affiliations:** a Department of Allergy, Hospital Universitario Ramón y Cajal, Spain; b Academic in Allergy and Immunology, Allergy Division, Former Head, Ramon & Cajal University Hospital, Madrid, Spain; c Department of Allergology and Pulmonology, University Children’s Hospital, Belgrade, Serbia; d Medical Faculty, University of Belgrade, Belgrade, Serbia; e Department of Pediatrics, University of Chieti, Chieti, Italy; f Clinica Pediatrica, Dipartimento di Medicina e Chirurgia, Azienda Ospedaliero-Universitaria, Università di Parma, Italy; g Pediatric Allergy Unit, Department of Woman, Child and Adolescent, Geneva University Hospitals, Geneva, Switzerland; h Department of Paediatric Allergy, Guy's and St Thomas' NHS Foundation Trust, London, UK; i Section of Immunology, HIV and Allergy, Department of Medicine, Clinical Hospital University of Chile, Chile; j Department of Paediatric Allergy and Immunology, Faculty of Medicine, Mersin University, Mersin, Turkey; k Allergy & Severe Asthma Service, St Bartholomew's Hospital, Barts Health NHS Trust, London, UK; l Allergy Unit and Research Group, Hospital Regional Universitario de Málaga, UMA-IBIMA-BIONAND, ARADyAL, Málaga, Spain; m Department of Pediatrics, University of Calgary, Alberta Children’s Hospital, Calgary, Canada; n Department of Medicine, University of Calgary, Calgary, Canada; o Allergy and Clinical Immunology Department, Centro Hospitalar Universitário Santo António, Porto, Portugal; p Allergy Unit, Meyer Children’s Hospital IRCCS, Florence, Italy; q Division of Allergy, Asthma and Immunology, Scripps Clinic, San Diego, California, USA; r Hospital Quironsalud, Bizkaia, Bilbao-Erandio, Spain; s Faculdade Israelita de Ciências da Saúde Albert Einstein School of Medicine, Hospital Israelita Albert Einstein, São Paulo, Brazil; t Clinical Immunology and Allergy and Immunology Division, University of São Paulo, São Paulo, Brazil; u Sorbonne Université, INSERM, Institut Pierre Louis d’Epidémiologie et de Santé Publique, AP-HP Sorbonne Université, Hôpital Tenon, Département de dermatologie et allergologie, Paris, France; v Division of Immunology and Allergy, Department of Chest Diseases, School of Medicine, Ankara University, Ankara, Turkey; w Department of Dermatology and Allergy Biederstein, Technical University of Munich, Faculty of Medicine and Health, Munich, Germany; x Hospital Italiano of Buenos Aires, Argentina; y Department of Internal Medicine, Seoul National University Bundang Hospital, Seoul National University College of Medicine, Seongnam, Korea; z Division of Allergy, Clinical Immunology and Rheumatology, Department of Paediatrics, Federal University of São Paulo, Brazil; aa Department of Allergy and Immunology, IIS-Fundación Jiménez Díaz UAM, Spain; ab Allergy Clinic, Department of Dermatology and Allergy, Copenhagen University Hospital-Herlev and Gentofte, Copenhagen, Denmark; ac Department of Clinical Medicine, University of Copenhagen, Denmark; ad University of São Paulo, Brazil; ae Department of Pediatrics, Faculty of Medical & Health Sciences, Tel Aviv University and Sheba Medical Center, Israel; af Hospital Regional Universitario de Málaga, Spain; ag Department of Rheumatology, University of Lodz, Poland; ah Allergy Centre, Wythenshawe Hospital, Manchester University NHS Foundation Trust and the University of Manchester, Manchester, UK; ai Instituto Nacional de Pediatría, Mexico City, Mexico; aj ASST Mantova, Italy; ak Clinical Hospital, University of Chile, Santiago, Chile; al Hospital Italiano de Buenos Aires, Argentina; am Department of Allergy and Clinical Immunology, Ajou University School of Medicine, South Korea; an Division of Allergology, University of Cape Town, South Africa; ao Center for Drug Safety and Immunology, Department of Medicine, Vanderbilt University Medical Center, Nashville, TN, USA; ap Division of Allergy and Immunology, Cincinnati Children’s Hospital Medical Center, Cincinnati, OH, USA; aq Department of Allergy, Ramón y Cajal University Hospital, Madrid, Spain; ar Division of Allergy, Department of Pulmonology, Allergy and Thoracic Oncology, University Hospital of Montpellier, Montpellier, France; as Desbrest Institute of Epidemiology and Public Health, UMR 1318 University of Montpellier - INSERM, France; at Translational Research in Pediatric Specialties Area, Division of Allergy, Bambibno Gesù Children's Hospital, IRCCS, Rome, Italy; au Drug Desensitization Center, Catalan Institute of Oncology, Barcelona, Spain; av Allergy Service, Bellvitge University Hospital, Barcelona, Spain; aw Department of Respiratory Medicine & Allergy, Homerton Healthcare NHS Foundation Trust, London, UK; ax Division of Respiratory Medicine, Third Department of Medicine, Teikyo University Chiba Medical Center, Chiba, Japan

## Abstract

Drug hypersensitivity reactions (DHRs) in children and adolescents are less common than in adults but can have serious consequences if mismanaged. Mislabeling children as drug-allergic due to incomplete diagnostic evaluations leads to unnecessary medication restrictions, increased healthcare costs, and suboptimal treatment choices. This Statement from the World Allergy Organization (WAO) provides evidence-based recommendations for evaluating and managing pediatric DHRs, emphasizing accurate diagnosis through *in vivo* and *in vitro* testing, risk stratification, and personalized approaches. Antibiotics, particularly β-lactams, and non-steroidal anti-inflammatory drugs (NSAIDs) are the most frequently implicated drugs, with non-immediate reactions, such as maculopapular exanthema, being the most common presentation. The document also addresses emerging concerns, including monoclonal antibody-induced anaphylaxis and drug-induced enterocolitis syndrome. It underscores the need for specialized care in allergy centers with expertise in pediatric populations and advocates for multidisciplinary programs to manage complex cases, such as chemotherapy hypersensitivity and perioperative drug allergy. By addressing diagnostic challenges and clinical uncertainties, this document aims to improve the management of DHRs in children, reduce mislabeling, and enhance patient outcomes worldwide.

## Introduction

### An overview of drug allergy in pediatric population—Epidemiology (Berges-Gimeno)

Drug hypersensitivity reactions (DHRs) in children and adolescents are a burden for patients, parents, and the health systems. The prevalence of self-reported DHRs by clinicians or parents ranges from 1% to 10.2%.^1^ After allergy work-up, only 4%–9% of those reported cases are confirmed as true allergic.^1^, ^2^, ^3^, ^4^ Therefore, a high number of children are likely currently inappropriately labeled as “drug allergic”.

There are known risks to carrying drug allergy labels in the pediatric population which has been documented in many studies revealing use of broader spectrum antibiotics, longer hospital stays, increased health care costs, and hospital readmittance.^5^^,^^6^ One recent study revealed that children admitted with pneumonia with a penicillin allergy label were more likely to develop respiratory distress compared to those that did not.^7^

Prioritizing the diagnosis and management of drug allergies in the pediatric population should be one of the main strategies to address this serious public health problem.

DHRs are clinically classified as immediate drug hypersensitivity reactions (IDHRs), those that occur within 1–6 h after drug administration or non-immediate reactions (NIDHRs), those that occur after 6 h up to days from taking the drug.^8^ NIDHRs such as maculopapular exanthema (MPE) are the most common clinical finding of DHRs in children.^1^, ^2^, ^3^, ^4^ Drugs most commonly implicated in drug hypersensitivity among the pediatric population are antibiotics (particularly β-lactam antibiotics), followed by NSAIDs, probably related to high prescription rates worldwide.^1^, ^2^, ^3^, ^4^ NSAIDs and betalactams (BLs) are the most frequent elicitors of drug*s*-induced anaphylaxis (DIA) in children but there are some geographical variations worldwide.^9^, ^10^, ^11^, ^12^ Recently, there has been an increase in hospital admission rates for DIA^9^ and monoclonal antibodies have emerged as a new leading category of drugs associated with DIA.^13^

The overall objective of this consensus document is to provide evidence-based recommendations to optimize pediatric patients care from diagnosis to management of drugs hypersensitivity reactions. In addition, it aims to address practical clinical questions and discuss key areas of uncertainty.

### Mechanisms and clinical responses to drugs in pediatric population: breaking views (Guzman-Melendez)

Challenging aspects of DHRs in the pediatric population include age-related differences in drug metabolism and natural history, the impact of co-factors such as frequent viral infections during childhood, and the variability in clinical presentations, biomarkers, and risk stratification strategies.^14^

Additionally, we highlight the critical need for dedicated facilities and frameworks to perform *in vivo* diagnostics, drug provocation tests, and drug desensitization, ensuring tailored approaches that address the needs of patients and their referring physicians.^15^

#### Developmental and pharmacological aspects

Drug responses in children and adults share many similarities, but perceived differences often reflect the lack of robust pediatric studies in 20th-century literature.^16^

Age-related differences in pharmacokinetics affect absorption, distribution, metabolism, and clearance (**see** Supplemental Table 1), leading to variations in plasma concentrations of parent drugs and metabolites.^17^, ^18^, ^19^

Adverse drug reactions (ADRs) may arise from metabolic differences. For instance, the increased use of trimethoprim-sulfamethoxazole (TMP-SMX) in pediatrics between 2005 and 2009 was associated with a significant rise in DHRs, including Stevens-Johnson Syndrome (SJS) and toxic epidermal necrolysis (TEN), likely linked to the biotransformation of both components.^20^ A potential cytotoxic biomarker of TMP-SMX, an N-acetyl-l-cysteine-TMP-SMX adduct detected in urine, has been described in pediatric patients.^21^

This increase in reactions may partly reflect greater drug use across all age groups. In children, however, pharmacokinetic differences—such as variations in absorption, distribution, metabolism, and excretion (Supplemental Table 1)—may further contribute to the higher incidence of DHRs compared with adults.

The intestinal microbiota also plays a role in drug metabolism, contributing to reductive and hydrolytic reactions.^22^^,^^23^ This microbiota can be influenced by factors such as delivery mode, antibiotic use in the mother or neonate, and, most notably, diet.^18^ The absence of a normal microbiota and the immaturity of bacterial enzyme systems may significantly impact drug metabolism.^24^

#### Mechanisms of drug allergy

DHRs are traditionally characterised by immune mechanisms mediated by drug-specific antibodies or activated T-lymphocytes. The revised DHR classification of the European Academy of Allergy and Clinical Immunology (EAACI) identifies 7 types of reactions. Traditional Gell and Coombs classifications (Type I–IV) remain predominant, while new types—Type V (epithelial barrier defects), Type VI (immune dysregulation related to metabolites), and Type VII (direct chemical responses)—address additional mechanisms.^25^ For example, Type V includes drug-induced enterocolitis syndrome (DIES), primarily described in children, and Type VII encompasses aspirin-exacerbated respiratory disease (AERD), rare in childhood.^26^

Drugs typically act as haptens, binding carrier proteins to elicit immune responses (eg, beta-lactams).^27^ Some drugs require metabolism to form reactive intermediates (pro-haptens, eg, TMP-SMX). Others bypass conventional immune pathways through the pharmacological interaction (PI) concept, directly binding T-cell receptors (TCR) in human leukocyte antigen (HLA)-restricted contexts.^28^

Drug-related cytotoxicity can amplify immune responses (danger concept), and some non-reactive drugs can act immunogenically via reversible, non-covalent binding to TCRs, which needs the expression of a particular HLA to be activated. This PI mechanism activates memory and effector T cells without prior sensitization or drug metabolism, bypassing the normal immune response. The interaction occurs when the drug binds to specific TCRs with sufficient affinity to trigger a signal, which is enhanced by major histocompatibility complex (MHC) interaction, leading to T-cell proliferation. Importantly, the PI mechanism does not involve B cells.^28^

In children, viral infections complicate DHR diagnosis, as viral exanthems may mimic hypersensitivity, particularly during Epstein-Barr virus (EBV) infections. Aminopenicillins, such as amoxicillin, often cause exanthems in this context, possibly through altered immune regulation or drug metabolism. While the exact mechanism remains unclear, it is plausible that viral infections like EBV may alter the antigenic expression of the drug or its metabolites, or disrupt immune regulation, thereby precipitating skin reactions like those seen with amoxicillin.^29^, ^30^, ^31^, ^32^, ^33^

The presumed mechanism behind a DHR influences the investigation method and diagnosis. Viral infections, common in childhood, are both a differential diagnosis and risk factor for DHRs, but in cases of skin rashes associated with beta-lactam treatments, less than 10% are confirmed as true DHRs.^29^^,^^30^ On the other hand, studies reveal some children retain penicillin sensitization into adulthood, especially following severe rashes during EBV infection.^34^, ^35^, ^36^

Confirmed DHR prevalence in children is lower than in adults, reflecting differences in drug exposure and diagnostic accuracy. Drug allergy labels in childhood are frequently unverified, leading to misdiagnosis that persists into adulthood.^37^

Viral reactivation also plays a pathogenic role in drug-induced hypersensitivity Syndrome (DIHS), also known as drug reaction with eosinophilia and systemic symptoms (DRESS).^38^ This condition is characterized by delayed onset, paradoxical worsening of symptoms after drug withdrawal, and unexplained cross-reactivity to structurally diverse drugs. DIHS/DRESS is a cell-mediated DHR, involving mechanism IVb, T2, and eosinophils contribute to allergic inflammation^25^ Herpes viruses (HHV-6, Epstein-Barr, cytomegalovirus, and HHV-7) can reactivate sequentially in DIHS/DRESS, triggering cross-reactive memory T-cells in a manner similar to graft-versus-host disease (GVHD).

DHRs may be linked to host genetic factors that enhance the immune response to drug compounds. Specific DHRs are more prevalent in populations with certain genetic alleles. For example, abacavir-induced DHRs are common in HIV-positive patients with the HLA B∗5701 allele, and its prescription requires a negative test for this allele. In the Han Chinese population, HLA B∗5801 is associated with allopurinol-induced severe cutaneous allergic reactions.^39^, ^40^, ^41^ Recent advances in understanding the genetic risk and immunopathogenesis of severe cutaneous adverse reactions (SCARs) have revealed strong associations between HLA class I risk alleles and drugs causing SCARs, including SJS/TEN and DRESS/DIHS, though not yet for acute generalized exanthematous pustulosis.^42^

NSAID hypersensitivity reactions exhibit selective immune-mediated phenotypes unrelated to cyclooxygenase (COX) inhibition.^43^ Cross-intolerant phenotypes are linked to metabolic imbalances, resulting in increased leukotriene production and the release of mast cell and eosinophil-derived mediators following use of non-selective NSAIDs. NSAIDs can also act as co-factors, exacerbating food-dependent, co-factor-induced urticaria/angioedema or anaphylaxis, and are commonly associated with non-specific lipid transfer protein (nsLTP) allergy.^44^^,^^45^

Understanding these mechanisms is crucial for personalizing diagnoses, treatments, and patient risk stratification.

#### Clinical aspects and risk stratification

The most common DHR manifestations in children are maculopapular exanthemas, urticaria, and angioedema.^46^ Systemic reactions and severe anaphylaxis are rare, accounting for only 5% of pediatric anaphylaxis cases.^47^ Anaphylaxis has a clearer causal link to β-lactams and NSAIDs, with intravenous drug administration strongly associated with immediate reactions.^48^^,^^49^

Unfortunately, suspected DHRs, particularly MPE, are less commonly thoroughly studied for diagnostic confirmation or exclusion.^28^

In adults, drug allergy is more common in females, but this is not observed in children.^50^ Children with chronic conditions and higher drug exposure are at increased risk.^51^

Antiepileptic drugs (AEDs) are primarily associated with delayed hypersensitivity reactions in children, typically self-limited but sometimes severe or life-threatening.^52^ Risk factors include prior AED reactions, viral infections, concomitant AED use, genetic factors, drug structure, and age-related drug metabolism differences. Children under 5 have an elevated risk of SCARs, with carbamazepine-associated DIHS/DRESS more prevalent in children than adults.^53^

#### Optimizing allergy care through a dedicated technical area for diagnostic and therapeutic procedures in allergy

While understanding the clinical and mechanistic aspects of DHRs is essential for accurate diagnosis and personalized treatment, it is equally crucial to ensure access to specialized areas with the necessary infrastructure and expertise.^15^ Another recent World Allergy Organization (WAO) Statement highlighted how a dedicated Technical Area is fundamental to excellent and safe care in drug allergy.^15^, ^54^ This current WAO Statement aims to provide a detailed, practical approach to integrating such a Technical Area into pediatric allergy care, and the full document is available in Supplemental Appendix 1.

## Allergy diagnostic workup

### *In vivo* diagnostic tests (Berges-Gimeno)

An allergy diagnosis based on categorization of drug hypersensitivity reactions through phenotypes, endotypes and biomarkers is crucial to the optimal management of patients with suspected of DHRs.^55^

A detailed clinical history is the first step of allergic work up. The history is often taken from parents or caregivers who may be biased by their subjectivity and interpretation. Older children and adolescents should be encouraged by the clinicians to provide key information about the reaction. It can be difficult to determine if symptoms are caused by an allergic mechanism or by an infection. Previous studies suggest that a suggestive clinical history is not enough to make a diagnosis of drug allergy.^46^ However, an allergy history focused on clinical characteristics, danger sign,s and timing of onset of reactions, is useful for identification of phenotypes and especially high-risk severe phenotypes and therefore key for planning allergy testing.^55^

It is generally recommended to perform diagnostic testing 4–6 weeks after the reaction and in DRESS; they should be done >6 months after the reaction disappears.^56^

*In vivo* diagnostic tests can be performed earlier in patients who need urgent rescheduling (antineoplastic agents, general anesthetics). Skin tests (ST), such as skin prick (SPT) and immediate reading of intradermal tests (IDT) are considered biomarkers for immediate reactions. Patch test and delayed reading of IDT may be useful in the diagnosis of non-immediate reactions. Non-irritanting concentrations established in adults have been extrapolated to determine STs concentrations in the pediatric population.^57^ Reliable skin tests are validated only for a few drugs.^57^ STs can be painful and may have suboptimal diagnostic value,^46^^,^^57^^,^^58^ and further research is needed to clarify their usefulness in children in clinical practice. Patch test sensitivity has been described as low,^59^ but are valuable in the work-up of MPE, acute generalized exanthematous pustulosis (AGEP), and DRESS.^56^ STs are generally safe; few systemic reactions after STs have been reported in children.^60^

Drug provocation test (DPT) is considered the gold standard to establish or exclude drug hypersensitivity.^46^ DPTs are not risks-free, so the benefit and risk for each patient must be carefully evaluated before performing the procedure. Risk stratification should be done based on the severity, mechanism of reactions, patient comorbidities, and safety conditions where the test is performed (site equipment and medical supervision).^61^, ^62^, ^63^, ^64^, ^65^ DPT without previous skin testing appears safe for diagnosing children with low-risk NIDHRs.^64^^,^^66^

DPTs should not be performed in patients who have experienced life-threatening reactions: severe anaphylaxis, vasculitic syndromes, SCARs, specific organ manifestations, such as blood cytopenia, hepatitis, nephritis, and pneumonitis.^46^^,^^64^ *In vivo* testing should be performed by specialists experienced in drug allergy in children and in a clinical setting where immediate clinical support is available to treat potential severe allergic reactions (see Supplemental Appendix 1).^15^^,^^46^^,^^64^

### *In vitro* diagnostic test (Mayorga)

The choice of *in vitro* tools for evaluating DHRs will be based on patient's phenotypes and endotypes with specific biomarkers, including IgE, T-cell, basophil activation, inflammatory mediators, or cell phenotype (see Supplemental Table 2). In IDHRs, immunoassay and basophil activation test (BAT) are the most widely used *in vitro* methods for identifying the responsible drug although with a wide range in sensitivity and specificity and being only available for limited number of drugs/agents (see Supplemental Table 3, and Supplemental Table 4). In children, drug-specific-IgE determinations by immunoassays show low sensitivity.^67^ BAT seems useful in diagnosing neuromuscular blocking agents (NMBA) allergy.^68^, ^69^, ^70^, ^71^ However, there are contradictory opinions about its role as a supplementary test to ST or about its correlation with DPT in DHRs to antibiotics.^72^, ^73^, ^74^ Measurement of tryptase or histamine during the acute phase, important inflammatory mediators in IDHRs, allow the assessment of mast cell degranulation with variable sensitivity and specificity, although they are not useful to identify the responsible drug.^75^, ^76^, ^77^

In NIDHRs, studies are mainly based on lymphocyte transformation test (LTT) or ELISpot (Ennzyme-linked Immunosorbent Spot), especially for SCARs where no other *in vivo* tests could be applied.^78^, ^79^, ^80^ However, they are still primarily used in the research and not commercially available in many areas. These tests have been mainly applied on antibiotics and anticonvulsant hypersensitivity.^79^^,^^81^ LTT in anticonvulsant hypersensitivity showed higher sensitivity compared to ST.^79^^,^^80^^,^^82^ Moreover, a modified LTT determining the expression of CFSElow (Carboxyfluorecein succimidil ester) showed a clear TH1 pattern (IFNγ+cells) in patients. With betalactams, although no positive H-thymidine-LTT was found in patients with positive DPT,^74^ others indicated its usefulness in NIDHRs to amoxicillin or amoxicillin-clavulanic acid.^83^^,^^84^ Moreover, the IFN-γ- and IL-4-ELISpot showed better results compared to LTT. Interestingly, the combination of IFN-γ and IL-4 assays produced higher positive drug-specific responses in both the acute and post recovery phases.^85^ (see Supplemental Table 3).

Since infectious diseases can act as important co-factors in children with NIDHRs,^86^ LTT could potentially assess sensitization to amoxicillin after detecting amoxicillin specific T memory lymphocytes in children with exanthema during EBV infection. These results can be helpful since *in vivo* tests may not be informative, particularly when DPTs are contraindicated due to the risk of reproducing the prior severe cutaneous adverse reaction.^83^^,^^87^, ^88^, ^89^, ^90^

## Evaluation and management specific drugs

### Beta-lactam antibiotics (Atanaskovic-Markovic)

Beta-lactam antibiotics (BLs), which include penicilins, cephalosporins, clavams, carbapenems, and monobactams, are the most common cause of DHRs in children.^91^^,^^92^

DHRs to BLs are classified according to timing between the start of treatment until symptoms appear into IDHRs, occurring within 1 h and up to 6 h after the drug intake and IDHRs, occurring at any time greater than 6 h, often starting from 3 to >12 days, or on the first day in re-exposures.^93^^,^^94^

The clinical entities of IDHRs range from mild/benign reactions such as urticaria and/or angio-edema to severe/life-threatening reactions such as anaphylaxis/anaphylactic shock.^91^^,^^92^

The clinical entities of NIDHRs extend from mild/benign reactions such as MPE or urticarial exanthema (UE) or serum sickness-like reactions (SSLR) to SCARs.^91^^,^^92^ SSLRs are characterized by presence of rash, arthralgia/arthritis and often fever. Amoxicillin is the most common implicated in pediatric population.

The 2 main goals of the diagnostic workup in subjects reporting DHRs to BLs are: to confirm or exclude allergy to the BLs concerned and in case of allergy diagnosis to find safe alternatives, particularly among other BLs.^94^

Assessment of DHRs to BLs involve: complete and precise clinical history about possible allergic reaction, which include a precise description of the morphology and chronology of the reaction, the drug/s involved, the time interval between drug administration and the reaction, as well as reliable *in vitro* and *in vivo* tests.^91^^,^^94^

In IDHRs the allergy workup should ideally be carried out 3–6 weeks after the complete resolution of all clinical symptoms as well as the clearance from the circulation of the incriminated betalactams and anti-allergic medications.^94^

*In vitro* diagnosis of IDHRs to BLs include quantification of serum drug specific IgE (sIgE) measurements. Immunoassays are available for amoxicillin, ampicillin, benzylpenicillin, phenoxymetilpenicillin, and Cefaclor. The sensitivity of assays is rather low and variable (0%–50%) and it correlates with the severity of the reactions. It is advisable to perform sIgE in addition to STs in severe reactions (high-risk patients) in order to improve the sensitivity of the allergy workup and thus reduce the need for DPTs.

Quantification of tryptase or BAT can be also useful in severe IDHR. BAT can be used in evaluating immediate reactions for which no immunoassays are commercially available, such as clavulanic acid and cefazolin.^91^^,^^92^^,^^94^

The recommended algorithm for DHRs to BLs (Supplemental Fig. 1) includes skin prick testing (SPT) and if it is negative, then intradermal test (IDT) with culprit drug is performed. Highest nonirritating concentrations recommended for STs^94^ are shown in Supplemental Table 5. If the results of STs are negative, DPTs should be considered.

In the case of mild/benign (non-severe) IDHR with skin symptoms only, the starting dose for DPT should not exceed 1:10 of the single target dose. The increasing doses at 30 min intervals are given until the full single dose is reached. In cases of anaphylaxis, starting dose should not exceed 1:100 of the single target dose.^64^ It has been suggested to skip skin testing in children with mild/benign IDHRs, so that DPTs can be done directly.^92^

In NIDHR, the allergy workup should ideally be carried out at least 4 weeks after the disappearance of cutaneous adverse drug reactions.^94^ *In vitro* diagnosis of NIDHRs to BLs include IFN-γ ELISpot and LTT in adults but with limited use in children.^91^^,^^92^^,^^94^

The recommended algorithm for mild/benign NIDHRs to BLs (Supplemental Fig. 2) includes DPT with the culprit drug with skipping skin testing. The starting dose is a full dose or 1:10. The observation time should be minimum 1–2 h after the last dose, depending on the individual risk for the patient, because severe reactions can occur during this time interval.^64^ There is no consensus for performing DPTs in NIDHRs. Some protocols have proposed to administer the initial full dose all at once, or by a graded challenge with incremental doses.^64^^,^^66^^,^^92^ Some groups perform prolonged DPT over 2–4 days, with varying washout periods between the initial dose and the next administration; however, this is a controversial practice, and it remains unclear whether prolonged protocols are superior to a single-day approach.^64^^,^^92^^,^^94^^,^^95^

Patch tests with the culprit BL can be conducted after 4 weeks but at least 6 months in DRESS after the disappearance of cutaneous adverse drug reactions, and should be read in 48 h and 96 h. In case of patch test negativity, the BL should be initially tested with the highest dilution for IDT. DPT is contraindicated in SCARs with the highly suspected drugs.^91^^,^^92^^,^^94^

Children who report mild IDHRs to BLs and have a pressing need for another alternative BL can be evaluated by STs with alternative BLs not sharing similar or identical side chains with the culprit drug and in case of negative results, can undergo graded challenges with the alternative BL concerned.^94^^,^^95^ The cross-reactivity between beta-lactams is thought to be caused by the common R1 side chain.^96^

In children with mild NIDHRs to BLs who require an alternative BL, when there is no time to perform an allergy workup with the culprit drug, giving a full dose of a structurally non-related BL under close surveillance should be considered.^94^^,^^95^ The cross-reactivity between penicillin and carbapenems seems negligible in these cases (<1%).^94^^,^^95^

### Non-betalactam antibiotics (Mori)

Non-betalactam antibiotic (NBLA) can trigger both IDHRs or NIDHRs although less frequently than BLs. Among NBLA trimethoprim-sulfamethoxazole and macrolides antibiotics are more frequently implicated in children.^97^ IDHRs range from urticaria to anaphylaxis and occur from 1 up to 6 h from the last drug intake. NIDHRs type reactions occur after 6 h up to days from the start of therapy being mild in 90% of cases such as maculopapular or morbilliform exanthemas and resolving spontaneously once the offending drug has been withdrawn. Only between 5% and 10% of these reactions may results in more SCARs. Moreover, NBLAs may provoke phototoxic or photoallergic reactions that are not seen with BLs being unique and characteristic of this group of drugs in particular of fluoroquinolones, sulfamides and tetracyclines.^98^^,^^99^ The underline pathomechanisms of DHRs to NBLAs vary according to their different chemical structure, metabolism and genetic predisposition (Table 1). For example, fluoroquinolones can provoke immediate reactions at first use without previous sensitization. This type of reaction is not driven by IgE, but it can be due to the activation of mast cells after binding to Mas-related G-protein-coupled receptor member X2 (MRGPRX2).^25^

**Table 1:** Non-betalactams Antibiotics characteristics, cross reactivity and desensitization protocols.

Non-betalactams antibiotics	Chemical structure	Prevalence/incidence	Pathomecanisms	Clinical manifestations (most frequently associated)	Cross-reactivity	Desensitization
**Sulfonamides (TMP-SMX)**	Sulfonamide (SO_2_NH_2_) moiety	P: 0.5% for 0–9 yr; P: 2.2% for 10–19 yr In HIV patients ten-fold increased risk	SMX oxidation to NO-SMX HLA-B∗13:01(Thailand) T cells, immune complex diseases, immune-mediated hepatotoxicity	MPE SCARs	Not documented between sulfonamides and drug with sulfonamide-like structure (dapsone; furosemide; celecoxib) ∗Exception: 1.SMX-sulfasalazine 2.SMX-amprenavir 3.SMX-fosamprenavir	Oral desensitization protocols (duration from 4 h to 4 weeks)^105^
**Macrolides**	Macrocyclic lactone ring with 12 (methymycin, picromycin), 14 (erythromycin, troleandomycin, roxitromycin, dirithromycin, clarithromycin), 15 (azithromycin), 16 (spiramycin, josamycin, midecamycin) carbon atoms with 1 or more sugar attached	P: 0.4% for 0–9 yr; P: 0.7% for 10–19 yr The rate of positive DPT ranges between 2 and 7.5%^106^ Clarithromycin anaphylaxis (1 per million)^107^	IgE-mediated T-cell mediated	Urticaria^108^ Urticaria/angioedema Anaphylaxis (clarithromycin, azithromycin, erithromycin) MPE	Spiramycin – erythromycin Clarithromycin- azithromycin In general, the majority of patients can tolerate an alternative macrolide particularly a macrolide with a different number of carbon atoms	Oral clarithromycin desensitization^109^ 13 steps 4 different concentrations (incremental doses: 0.03–0.09-0.22–0.47-1-2-4-8-16-32-64-128-253 mg) Oral azithromycin desensitization^109^ steps 4 different concentrations (incremental doses: 0.03–0.09-0.2–0.45-1-2-4-8-16-32-64-189-314 mg)
**Glycopeptides (vancomycin)**	Composed of glycosylated cyclic or polycyclic nonribosomal peptides	I: 5–14% of treated children^110^^,^^111^	Direct mastcells degranulation through MRGPRX2 HLA A∗32:01^112^	VIR (flushing; erythematous rash, pruritus) Anaphylaxis SCARS (DRESS) LABD, FDE acute interstitial nephritis	Vancomycin-teicoplanin (adults) Teicoplanin does not activate MRGPRX2 so it can be tolerated in case of VIR	VIR: Antihistamine pretreatment; infusion rate reduced by ≥ 50% IgE-mediated: I.V. desensitization (0.0001 mg/ml with a ten-fold increases every 10 min up to 10 mg/ml)^113^
**Aminoglicosides**	Hexose ring with amino group substituents to which various amino sugars are attached via glycosidic linkages Two groups: Streptidine group (streptomycin); Deoxystreptamine group (gentamicin, tobramycin, neomycin, amikacin, kanamycin, plazomicin, paromomycin)	11.5% sensitized to neomycin among asymptomatic schoolchildren^114^	IgE-mediated T-cell mediated	Allergic contact dermatitis (neomycin) Urticaria Anaphylaxis MPE, ED, DRESS	>50% In particular all deoxystreptamine-containing ami- noglycosides are contraindicated if a patient has a known hypersensitivity to another deoxystreptamine-containing aminoglycoside, but they can probably tolerate streptomycin^115^^,^^116^ Patients with anaphylaxis to topical or systemic neomycin should not be vaccinated with neomycin-containing vaccines	I.V. Tobramycin desensitization 0.001 mg dose doubling the dose every 10 min up to 16 mg; cumulative dose reached of 80 mg in 8 h Inhaled Tobramycin desensitization (0.3–0.6-0.9–1.2-1.5-3-6-12-24-48-96-150-200-250-300 mg: each dose in 50 ml of normal saline-via nebulizer every 2 h)
**Tetracyclines**	Share a naphthacene core with similar Side groups (Doxycycline, minocycline, and tetracycline)	P: 4.2%^117^	T cell-mediated	SCARs DRESS to Minocycline (pneumonitis, myocarditis liver and renal involvement)	Cross-reactivity among tetracyclines is not well defined Possible between: Minocycline/doxycycline minocycline/tigecycline	Oral Doxicycline 12 steps protocol (0.00001–0.0001-0.001–0.01-0.1-1-2-4-8-12-25-50 mg every 30 min) cumulative dose 100 mg Oral Minocycline 14 steps protocol (0.01–0.02-0.04–0.08-0-16-0.32-0.64-1.28-2.56-5-10-20-40-60 mg every 15 min) cumulative dose 100 mg^118^
**Quinolones**	Made of a bicyclic skeleton with carboxylic acid and ketone groups (ciprofloxacin, moxifloxacin, levofloxacin, ofloxacin, delafloxacin, and gatifloxacin)	I (Spain): 0.53% in 2005–5.96% in 2009 Anaphylaxis incidence in schoolchildren: 1–1000 (ciprofloxacin)	IgE-mediated T cell-mediated Dose-dependent direct mastcells degranulation through MRGPRX2 (moxifloxacin)	Urticaria Anaphylaxis MPE SCARs	It is high between first- and second-generation quinolones Levofloxacin may be a safe alternative in cases of reaction to first-, second-, or fourth-generation quinolones^119^	IV ciprofloxacin desensitization starting dose 0.00001 mg in 15 min, tenfold increases. From 0.01 mg twofold increases every 15 min^120^
**Antituberculosis drugs**	INH: Isonicotinic acid hydrazide RIF: Semi-synthetic agent of the group of rifamycin antibiotics PZA: a Synthetic pyrazine analogue of nicotinamide EMB: 2 constitutionally symmetrical chiral centers in its structure and exists in 3 stereoisomeric forms, the enantiomeric pair (+)-(S,S)- and (−)-(R,R)-ethambutol, along with the achiral stereoisomer called meso-form	I:0.1–20% (children and adults) Frequency increases with age: 2.3% 0–19yr 8.4% ≥ 60yr^121^	T cell-mediated	MPE SCARs	–	Oral RIF: 13 steps protocol (0.19–0.038-0.075–0.15-0.3–0.6-1.2-2.4-4.8-9.5-19-38-75 mg every 15 min) cumulative dose of 150 mg^122^ INH EMB^123^
**Clindamycin**	Is a derivative of lincomycin with a 7(S)-chloro-substitution of the 7(R)-hydroxyl group	I: 0.47% (adults and children)	T cell-mediated Cytochrome P450 drug-metabolizing enzyme polymorphisms (CYP2C19 and CYP2C9)	MPE DRESS^124^ (SDRIFE AGEP)	There are no data on potential cross- reactivity of clindamycin with other antibiotics	Rapid oral desensitization: 9 step-approach with 30 min interval (0.005–0.05-0.5-5-10-20-40-80-150 mg) over 4.5 h until a cumulative dose of 300 mg^125^
**Linezolid**	Oxazolidinone, a heterocyclic molecule with a nitrogen and oxygen in a 5-membered ring bridged with a carbonyl group	1.7% of treated patients (adults and children)^126^	IgE-mediated T cell-mediated	Urticaria Angioedema Anaphylaxis Interstitial nephritis DRESS	There are no data on potential cross- reactivity of linezolid with other antibiotics	Three different solutions containing 0.02–0.2-2 mg/ml concentrations of Linezolid were prepared, the doses of Linezolid (0.03–0.07-0.15–0.3-0.6–1.2-2.3-4.7-9.4-18.75-37.5-75-110 mg) were given in a total of 13 steps (2 doses from the first concentration, 4 from the second and 7 from the third one) within 4 h and 15 min
**Metronidazole**	A nitroimidazole with structural similarities to tinidazole, clotrimazole, ketoconazole, miconazole, and albendazole	–	IgE-mediated T-cell mediated	Urticaria Anaphylaxis SCARs (adults)	A potential for cross-reactivity exists between metronidazole and other imidazoles, such as tini- dazole, clotrimazole, ketoconazole, miconazole, and alben- dazole given structural similarities	I.V. metronidazole desensitization with a 14 steps protocols; doses administered at 10–15 min intervals (0.005–0.015-0.05–0.15-0.5–1.5-5-15-30-60-125-250-500-2000 mg) (adults)^127^

TMP = trimethoprim; SMX Sulphametoxazole; P = prevalence; I= Incidence; MPE = maculopapular exanthemas; SCARs = severe cutaneous adverse drug reactions; SMX = sulfamethoxazole; DPT = Drug Provocation Test; VIR=Vancomycin infusion reaction; DRESS = drug reaction with eosinophilia and systemic symptoms; LABD = Linear IgA bullous dermatosis; FDE = fixed drug eruption; SDRIFE = drug-related intertriginous and flexural exanthema; Isoniazid = INH; RIF = rifampicin; PZA = pyrazinamide; EMB = ethambutol

The reported rates of confirmed DHRs to NBLAs is greatly variable from 0.2% to 27.2% in children (Table 1).^97^ The rate of NBLAs allergy is higher if based only on clinical history. In a previous study it was of 15.6% when children were diagnosed according to the anamnesis, but this percentage dropped to 4.7% when children were evaluated with intradermal test and/or drug provocation test.^100^ Some major limitations affect the diagnostic work-up of DHRs to NBLAs, in particular skin testing is not sufficiently sensitive and consequently is poorly predictive of a subsequent reaction. Moreover, skin tests are not standardized, and in high concentrations can result in false-positive reactions due to irritating properties of the drug. Non-irritating skin test concentrations have been proposed for some NBLAs, but have not yet been confirmed for children (Supplemental Table 4).^101^^,^^102^

*In vitro* tests can be used in immediate reactions, such as serum-specific IgE assays and BAT but today remain experimental for non-beta-lactam antibiotics.^103^^,^^104^ Other research tools, such as LTT and flow cytometric lymphocyte activation test (LAT), can be diagnostic aids in some T cell-mediated reactions.

DPTs remains the gold standard for diagnosis but it cannot be performed in case of SCARs, hematologic/autoimmune diseases or severe immediate reactions for ethical reasons due to risk for life-threatening reactions.

At last, data on the potential for cross-reactions between and within NBLA classes are limited, especially in children. In case of not safe or equally effective non-betalactam drug alternative, few desensitization protocols have been published in children (Table 1).

### Non-steroidal anti-inflammatory drugs (NSAIDs) (White)

As with adults, the diagnosis and management of hypersensitivity to NSAIDs can be challenging in the pediatric population. The lack of skin testing/*in vitro* testing and the reliance on challenges create most of the difficulty. NSAIDS are the most widely consumed medications in pediatrics, thus it is not surprising that NSAID hypersensitivity is second only to beta lactams in reported allergy in this group.^128^ ^.^ However, NSAIDs are the leading case of DHRs in adults and children in Latin America. Yet, confirmed hypersensitivity to BLs is less than 10% with NSAID hypersensitivity confirmed in approximately 20% of children.^129^, ^130^, ^131^, ^132^ Worldwide, ibuprofen and paracetamol are the most commonly reported drugs to trigger hypersensitivity.^129^

The 2 primary categories of reactions are related to the pharmacologic activity of the NSAID on cyclooxygenase-1 (COX-1) or the specific NSAID leading to what is likely immunologic recognition. The former generally does not cause anaphylaxis, whereas the latter might meet criteria for anaphylaxis but patients in either category might present similarly with cutaneous and respiratory symptoms among other findings.^43^^,^^133^ In adults, the NSAID reaction is generally categorized as NSAID-exacerbated respiratory disease (N-ERD) if primarily a respiratory reaction in the context of asthma and nasal polyps, NSAID-exacerbated cutaneous disease (NECD) if the reaction is a cutaneous exacerbation in the context of chronic spontaneous urticaria or single NSAID if the reaction occurs exclusively with 1 agent but other COX-1inhibitors are tolerated.^134^ N-ERD is extremely uncommon in the pediatric population but has been reported.^135^ If the reaction occurs with several COX-1 inhibitors, then the term single NSAID-induced urticaria, angioedema, and anaphylaxis (NIUAAA) is the best term to describe the breadth of reactivity the patient might experience, although true anaphylaxis is rare in this category. NSAIDs may also be involved in hypersensitivity reactions as a co-factor.^136^ A 2018 position paper from the European Academy of Allergy and Clinical Immunology and the European Network on Drug Allergy (EAACI/ENDA) outlined a simplified strategy categorizing reactions as cross-intolerant (all COX-1) or non-cross- intolerant (allergic).^43^

Although DPT remains the only diagnostic test and might be considered risky, the data suggest that most children will be cleared for NSAID use with a challenge; thus, it should be strongly considered (see Supplemental Fig. 3).^64^ Mori et al evaluated 629 children with NSAID reactions. Less than 20% had the NSAID hypersensitivity confirmed with challenge with the culprit drug.^129^ In children ≤2, DPT were positive in only 7%, pointing to a concomitant viral illness at the time of NSAID ingestion as the likely culprit. In this large series, most children were challenged with the culprit agent, thus when positive a cross intolerant or non-cross intolerant mechanism could not be determined. Although these data support DPT as an essential component of NSAID hypersensitivity evaluation, further study in the pediatric population is warranted.

### Vaccines (Caubet)

Hypersensitivity to vaccines constitutes a frequent issue for primary care physicians and allergists. In clinical practice, one of the most common situations is a patient known with an allergy to 1 vaccine constituent, who should receive a vaccine that may contain this constituent. Particularly, influenza vaccine and yellow fever vaccine have been incriminated to cause reaction in egg-allergic patients. Recently, it has been well demonstrated that egg-allergic patients do not have an increased risk for reaction to egg-based influenza vaccines.^137^ Thus, they can receive the influenza vaccine full dose, without special precaution. Regarding yellow fever vaccine, although several recent studies indicate that administration of this vaccine in egg allergic patients may be safe, it still needs to be confirmed if a similar approach can be proposed.^138^, ^139^, ^140^, ^141^ Currently, the first step is to confirm the egg allergy by skin test, specific IgE and/or an oral food challenge. If the egg allergy is confirmed, it is recommended to perform STs with the vaccine itself, and then to adapt the administration based on the STs results. Although rare, gelatin is also one of the most frequently incriminated vaccine constituents in allergic reactions to vaccine.^142^ In patients known to have an allergy to gelatin (by ingestion or after administration of a gelatin-containing vaccine), gelatin-free vaccine should be used. Otherwise, skin test with the vaccine itself should be performed before injection. Then, the vaccine can be given full dose (negative skin test) or in graded dose (positive skin test) after evaluation of the risk-benefit ratio. If a patient reacts to a gelatin-containing vaccine, it is also important to investigate for an underlying gelatin hypersensitivity, for future vaccine injection and to see if food containing gelatin should also be avoided. Of note, patients with alpha gal syndrome might also react to gelatin containing vaccine and should be investigated before vaccination.^143^^,^^144^ Recently COVID vaccine was administered largely, and rare anaphylactic reactions have been reported. It has been shown that most patients could be revaccinated safely.^145^ As for other vaccine, skin test to the incriminated vaccine and its components (ie, polyethylene glycol [PEG] and/or polysorbate 80 [PS80]) have been proposed in patients with immediate reactions to vaccine or to PEG/PS80.^146^ Of note, it has been shown that these skin tests might increase vaccination willingness.^147^

### Antiepileptic drugs (AEDs) (Kuyucu)

AEDs are the mainstay of epilepsy therapy but their use in non-epilepsy disorders such as psychiatric illnesses, addiction and pain disorders is rapidly increasing, especially among adolescents.

Epilepsy is the most common chronic neurological disorder affecting approximately 0.5–1% of children worldwide.^148^^,^^149^ Antiepileptic drugs (AEDs) are classified as aromatic and non-aromatic, or old and new generation.^150^^,^^151^ (see Supplemental Fig. 4). Sodium valproate (VPA), carbamazepine (CBZ), and lamotrigine (LTG) are the most commonly employed AEDs in children.^53^^,^^151^, ^152^, ^153^ Antiepileptics are among the most common causes of DHRs in children.^154^, ^155^, ^156^, ^157^, ^158^ New prospective pediatric studies revealed that 1.0–6.1% of children started on AEDs has developed DHRs, of which nearly 20% was severe cutaneous adverse reactions (SCARs) with a SCAR incidence is ranging between 1.0% and 1.1%.^53^^,^^152^^,^^159^ Although most cases of DHRs to AEDs, and especially SCARs, are related to aromatic AEDs, in the last few years there have been increasing number of reports with new generation non-aromatic AEDs, mostly levetiracetam (LEV).^53^^,^^152^^,^^160^, ^161^, ^162^, ^163^, ^164^, ^165^, ^166^

The major distinctive features of DHRs to AEDs in children, are the very strong associations to SCARs, especially SJS, (TEN, and drug reaction with DRESS;^71^^,^^167^, ^168^, ^169^, ^170^, ^171^, ^172^, ^173^, ^174^, ^175^ higher risk of drug-induced morbidity and mortality;^168^^,^^169^^,^^173^^,^^175^^,^^176^ and in addition an important contribution of drug interactions, metabolic derangements, and multi-ethnic cytochrome (CYP2C19) pharmacogenetic variations on DHRs development;^171^, ^172^, ^173^, ^174^, ^175^, ^176^, ^177^, ^178^, ^179^ and strong relation of severe delayed reactions to certain ethnicity-specific HLA alleles (HLA-B∗15:02-CBZ in Han Chinese and Southeast Asian, 0HLA-A∗31:01-CBZ in European, Chinese and Japanese and HLA-A∗01:01/HLA-B∗13:01-phenobarbital [PB] in Thai children and HLA-B∗57:01-CBZ in Europeans).^180^, ^181^, ^182^, ^183^, ^184^, ^185^ Young age, aromatic AEDs and multi-AED regimens, especially including VPA and LTG, are associated with an increased risk of DHRs to AEDs in children.^53^^,^^152^, ^153^, ^154^^,^^159^^,^^186^, ^187^, ^188^ (See Supplemental Table 5). Antiepileptic drug-induced hypersensitivity reactions present with a variety of clinical manifestations ranging from most frequent (80%–92%) mild skin rashes such as MPE and urticaria to more rare (5%–20%), but serious, and sometimes fatal, reactions such as SCARs.^53^^,^^71^^,^^152^, ^153^, ^154^^,^^165^^,^^189^ Agranülocytosis, thrombocytopenia, aplastic anemia, drug-induced liver injury (DILI) and pancreatitis are more rare, but AEDs are important in etiology.^154^^,^^187^^,^^190^, ^191^, ^192^, ^193^ (See Supplemental Fig. 5). VPA, which is a very commonly employed agent in pediatrics, especially has a significant risk of DILI and pancreatitis among children.^192^^,^^194^^,^^195^ Although, IgE-mediated reactions such as urticaria, angioedema and anaphylaxis are relatively rare (5–10%) when compared to T cell-mediated reactions, they may also occur with both old and new antiepileptics, especially with LEV.^163^^,^^189^^,^^196^, ^197^, ^198^, ^199^, ^200^, ^201^ Viral infections of childhood are common and may be easily misdiagnosed as an DHR to AED. However, it is uncertain whether they play a role or trigger DHRs to AEDs in children.^53^^,^^153^^,^^154^^,^^202^^,^^203^

All suspected children should undergo an algorithmic diagnostic workup to confim or exclude a real SPT, early and late reading IDT, patch test (PT), and DPT. *In vitro* tests useful in NIDHRs to AEDs are LTT, cytokine release and ELISpot assays, in addition to HLA analysis in special circumstances.^52^^,^^154^ Choice of the tests should be done according to endophenotypic classification. (Supplemental Fig. 5 and Supplemental Fig. 6). Patch test is the most commonly employed and useful skin test for diagnosis, and also for alternative drug choice, of NIDHRs to AEDs.^53^^,^^153^^,^^156^^,^^169^ It is a relatively safe method in also SCARs, when used in diluted concentrations.^53^^,^^153^^,^^154^^,^^204^, ^205^, ^206^, ^207^, ^208^, ^209^ Sensitivity changes according to culprit AED and clinical phenotype.^52^^,^^205^^,^^208^ Antiepileptics were the group most frequently associated with a positive PT result among all drugs in pediatric DHRs series and also in a systematic review of DRESS.^204^, ^205^, ^206^ In children the positivity rate and sensitivity of PTs with AEDs (in 1–30% in petrolatum concentrations) ranged from 20 to 90%, particularly found beneficial with CBZ, LTG, VPA, and for specific clinical manifestations such as DRESS and MPE, while the latter 2 presentations had nearly similar positivity rates in some series.^53^^,^^153^^,^^169^^,^^205^^,^^207^^,^^208^ (Supplemental Table 6). A recent systematic review including children and adults with SJS/TEN and a pediatric case series revealed that PTs with AEDs were also useful and relatively safe, with a systematic reaction is ranging from 0% to 3.7%.^205^^,^^209^ However, a high level of caution should be applied when testing in SJS/TEN and DRESS, especially in immunosuppressed population.^204^^,^^208^^,^^209^ The negative and positive predictive values and specifity of PTs with AEDs is still not clear, since comparison to gold standart DPT is lacking.^52^^,^^204^^,^^208^^,^^209^ If PTs are negative, IDTs with sterile preparations can be performed. A pediatric case series found a 10.6% positivity with AED IDTs.^153^ Intradermal tests with AEDs are recommended in immediate and mild delayed skin reactions but not in SCARs in children, especially SJS/TEN due to risk of flare ups.^52^^,^^153^^,^^210^ In selected cases of DRESS IDTs may be performed using diluted drug concentrations with long intervals.^52^^,^^154^ A retrospective multicenter study, including some children with SJS-TEN, showed that SPTs/IDTs, performed with higher dilutions than in other SCARs were not sensitive but did not reactivate neither rash beyond the test area nor relapse of SJS/TEN. However, SPT/IDT was not performed with AEDs in this study.^211^ In fact, optimal non-irritant skin test concentrations are not known for AEDs and testing should be accompanied with control testing.^52^^,^^154^^,^^205^^,^^212^ Since negative predictive value (NPV) of skin tests are largely unknown, a graded DPT should be performed with the culprit AED when skin tests are negative in mild index reactions. However DPT with the culprit is contraindicated in all severe reactions including SCARs.^52^^,^^71^^,^^154^^,^^170^^,^^205^^,^^210^ *In vitro* tests with AEDs can be especially useful in SCARs where diagnostic options are very limited. The sensitivities and specificities of different *in vitro* methods with AEDs in SCARs ranged between 58.4 to 88.9% and 86–95.8%, respectively in children.^213^, ^214^, ^215^, ^216^, ^217^ On the other hand, recent reports showed that ELISpot assay detected drug-specific IFN-γ-or IL-4 releasing cells in 73.9–82% of SCAR subjects during the acute phase, which pointed out that this method can be more sensitive than LTT performed in recovery phase and may also support culprit identification during acute reaction.^218^, ^219^, ^220^ Although in the last 2 decades there have been increasing numbers of revealed associations between HLA alleles and AED-DHRs, routine preventive HLA analysis is recommended for only CBZ. According to authorized consortiums, genetic testing for HLA-B∗15:02 is recommended for all CBZ-naive patients originating from populations where HLA-B∗15:02 is common before initiation of CBZ.^221^, ^222^, ^223^, ^224^, ^225^, ^226^, ^227^ Although it has been definitely shown that HLA-A∗31:01 was the strongest genetic predisposing factor for well-phenotyped CBZ-SCAR (espescially DRESS) and CBZ-DILI cases in Europeans, Chinese, and Japanese, routine testing for this allele has not been incorporated into preventive health policies yet.^225^, ^226^, ^227^, ^228^, ^229^

Any suspicion of DHRs to AEDs usually requires a change in therapy. Cross-reactivity (CR) among old and new aromatic AED, which occurs in 19–70% of patients, should be taken into account in choosing alternative, while CR of non-aromatic AEDs to aromatics or to other non-aromatics has not been reported.^230^, ^231^, ^232^, ^233^, ^234^, ^235^ On the other hand, some authors recommend that patients with mild rashes on low doses may be managed with only AED dosage reduction (eg, 50%) and observation, while severe reactions require a more rapid taper or abrupt cessation which should be done in a hospital setting.^236^ However, the risk of withdrawal seizures necessitates appropriate precautions to be taken. Desensitization is recommended in immediate or mild delayed reactions, if no alternative medication is available. It is contraindicated in patients who previously had SCARs or other severe reactions.^154^^,^^210^^,^^237^ In children there are few case reports about successful desensitization procedures in mostly delayed reactions with phenytoin, PB, LTG, oxcarbazepine, and VPA, most of them required weeks to months to achieve titration to the full dose.^238^, ^239^, ^240^, ^241^, ^242^, ^243^, ^244^, ^245^

### Radiocontrast media (Rebelo Gomes)

IDHRs and NIDHRs to non-ionic radiocontrast media (RCM) are reported in about 0.2–3% of adult patients. Recent work reporting on more than 288 million iopromide administrations found a hypersensitivity reaction rate for children of 0.0114%, significantly lower as compared with adults (0.0143%).^246^

A study evaluating the frequency and severity of acute allergic-like reactions to gadolinium-based contrast media (GBCM) in children reporting on 32,365 administrations refers an incidence of 0.06% with mostly mild or moderate (47% each) reactions.^247^

Several risk factors have been inconsistently identified but a previous reaction to RCM administration is considered the most important (Supplemental Table 7).^248^, ^249^, ^250^, ^251^, ^252^, ^253^, ^254^ DHRs to RCM include both IDHRs and NIDHR. Severe reactions tend to occur within minutes after administration.^248^, ^249^, ^250^, ^251^, ^252^, ^253^, ^254^

Most frequent presentations suggesting hypersensitivity are mild cutaneous reactions such as erythema, urticarial/angioedema, maculopapular exanthemas, and pruritus occurring in more than 70% of patients. Anaphylaxis and SCARs in children seem to be extremely rare.^248^, ^249^, ^250^, ^251^, ^252^, ^253^, ^254^

The diagnostic workup is based on the clinical history and it is only indicated for patients with a suspected hypersensitivity.^249^, ^250^, ^251^, ^252^, ^253^, ^254^ I*n vivo* and laboratory tests are used to document an allergic mechanism. Positive skin tests and *in vitro* tests indicating allergy are more common in immediate and in severe reactions.^249^, ^250^, ^251^, ^252^, ^253^, ^254^

A detailed clinical history is a paramount to decide the investigation.^249^, ^250^, ^251^, ^252^, ^253^, ^254^ (Supplemental Table 7) highlights the information to be inquired. At acute phase a tryptase measurement is recommended if anaphylaxis suspected.^251^, ^252^, ^253^, ^254^ The BAT is useful for the evaluation of immediate reactions. Sensitivities of up to 94% and specificities of 89–100% are reported. BAT results together with ST increase the possibility of a positive diagnosis.^251^, ^252^, ^253^, ^254^ The usefulness of BAT in immediate hypersensitivity (HS) to RCM and to GBCM has been demonstrated.^255^^,^^256^ In children this test would be a good starting point for diagnosis as it is minimal invasive and no risky.

In IDHRs skin prick tests and IDT with the culprit RCM and possible alternatives are important tools for diagnosis and should be performed up to 6 months after reactions. The commercial drugs are used undiluted for prick and a 1:10 dilutions used for IDT.^249^^,^^251^, ^252^, ^253^, ^254^

For NIDHRs both delayed reading of IDT and patch tests with undiluted RCM solutions can be used. Readings at 48 up to 120 h are recommended. Sensitivity of IDT seems to be higher but using both tests increases the performance.^249^^,^^251^, ^252^, ^253^, ^254^ In children starting with patch tests could avoid the need of more invasive and painful IDT.

Positivity rates of ST are described at 17% in patients with IDHRs and up to 52% for severe IDHRs with specificity of 96–100%. In NIDHRs, the positivity rate is around 26.^249^, ^250^, ^251^, ^252^, ^253^, ^254^^,^^257^

STs with alternatives are helpful for the selection of RCM for future procedures as cross reactivity is frequent especially in NIDHRs.^249^, ^250^, ^251^, ^252^^,^^257^^,^^258^

The negative predictive value of ST has been reported at 94% being higher for IDHRs. Recurrence rates of DHRs to ST-negative drugs were reported at 7% in immediate and 35% in NIDHRs.^249^, ^250^, ^251^, ^252^^,^^257^^,^^258^

The DPT is considered the gold standard for diagnosis but lacks standardization. A careful assessment of risk and benefice should be done; the main utility is to confirm the safety of an alternative RCM in patients with confirmed HS or with a previous severe reaction.^249^, ^250^, ^251^, ^252^, ^253^, ^254^^,^^257^, ^258^, ^259^

In patients with mild reactions and negative STs to the culprit a DPT with the culprit can be considered to exclude hypersensitivity.^249^, ^250^, ^251^, ^252^, ^253^, ^254^^,^^257^, ^258^, ^259^

Patients with previous severe anaphylaxis or SCARs even with negative STs should avoid future exposure and perform native CT or alternative imaging specially in the case of NIDHRs.^249^, ^250^, ^251^, ^252^, ^253^, ^254^^,^^257^, ^258^, ^259^ As DPTs are not consensual the re-administration of the RCM to the patient during a future needed radiological examination can also be considered and can actually be more rational instead of performing a DPT to prove safety.^250^, ^251^, ^252^, ^253^, ^254^

The diagnosis and management of patients vary among guidelines and according to the characteristics and severity of the previous reaction. Recommendations usually suggest the use of STs followed or not by DPTs although, in the case of very mild cutaneous reactions, a simple switch to a different RCM can also be a possible strategy.^251^, ^252^, ^253^, ^254^^,^^257^, ^258^, ^259^ An algorithm is proposed in Supplemental Fig. 7.

If the patient has to be exposed to a RCM before the allergy workup or in the case of patients with all STs negative an alternative drug should be used and premedication with antihistamines and/or corticosteroids considered.^251^, ^252^, ^253^, ^254^^,^^257^, ^258^, ^259^ There are not enough data on RCM cross reactivity to make strong recommendations on alternative selection in absence of ST. Some authors suggest that for the selection the classification of the RCM into non-ionic monomers (ioversol, iopromide, iomeprol, iopentol), ionic dimers (ioxaglate) and non-ionic dimers (iodixanol) should be taken into account and if the previous reaction occurred with a dimeric molecule a monomeric should be chosen and *vice versa*. The superiority of RCM substitution over premedication plus use of the same RCM is sound.^251^, ^252^, ^253^, ^254^^,^^260^, ^261^, ^262^, ^263^, ^264^

In pediatric patients, a change to a different RCM and premedication would also be advised in the case of high risk patients or previous severe reactions.^265^ Desensitization protocols were used in few adults in the case of urgent need.

Children have a lower incidence of DHRs to RCM. Most common clinical presentations are immediate or non-immediate mild cutaneous eruptions. Diagnosis and management are based mostly on experience with adult patients as specific pediatric guidelines are lacking. An approach that combines clinical history, STs and laboratorial tests to decide on re-administration with or without premedication should be effective to prevent recurrence.

## Severe cutaneous adverse reactions (SCARS)

### Drug reaction with eosinophilia and systemic symptoms (DRESS) (Caffarelli)

DRESS in children is a delayed type IVb hypersensitivity reaction to an increasing number of medications although the top drugs, especially antiepileptics and antibiotics, such as penicillins, thrimetoprin-sulfamethoxazole, and vancomycin have not changed much in the last 10 years.^168^, ^169^, ^170^^,^^215^ It is characterized by fever, rash, lymphadenopathy, and leukocyte abnormalities such as eosinophilia, lymphocyte activation, and organ involvement, most commonly hepatitis (Supplemental Table 8). Human herpesvirus-6, -7, cytomegalovirus, EBarrV, or parvovirus B19 replicate in 50% of children.^169^ Criteria for pediatric DRESS are the same as adults and are not validated in children (Supplemental Table 9 and Supplemental Table 10).^266^^,^^267^ DRESS can be mistaken, especially in children, for viral reactivation with eosinophilia and systemic symptoms that differ in clinical findings and timing of associated drug. The recovery can be protracted with relapse and/or autoimmune complications occurring in approximately 10%. Mortality rate is lower in children^168^^,^^170^^,^^268^^,^^269^ than in adults (1.7%–10%). Determination of HLA alleles associated with DRESS is not readily available. It may be useful for risk stratification and diagnosis such as HLA-A∗32:01 and vancomycin.^270^ Several studies have shown that patch test, IDT and LTT can be useful to ascertain a delayed hypersensitivity response to the culprit drug.^204^^,^^270^, ^271^, ^272^, ^273^ However, the accuracy of tests is unclear since the diagnosis of drug hypersensitivity was based on clinical history and not on drug provocation test, considered the reference standard. Provocation test is contraindicated for the risk of life-threatening symptoms.^169^^,^^204^^,^^270^, ^271^, ^272^ Patch tests are harmless. They are generally performed 6 months following acute onset or when the patient has been weaned off steroids and is stable. However they have been performed between 3 weeks and 3 months after the reaction, resulting positive in 61% of children,^169^ and 68% of adults.^204^ IDTs might trigger systemic reactions often confused with DRESS relapse.^272^ So, IDTs are done after negative results of patch test/prick test,^271^ 6 months after recovery, with reading after 24 and 72 h, and in some cases up to 96 h.^272^ Delayed positive skin prick test results consistent with a delayed hypersensitivity reaction have been reported at 24 h or >24 h but the interpretation is uncertain.^270^ However, according to practice parameters^270^ and guidelines^271^ skin prick test to the offending drug should be also performed with reading after 1–2 days. LTT is safe, but standardization lacks for several drugs, the process is difficult, results depend on drug and accuracy is lower in the acute phase.^273^ Sensitivity of LTT to amoxicillin in recovered children and adults is similar (75%).^215^ IFN-gamma and IL-4-ELISpot has also been assessed. Both ELISpot and LTT have a higher sensitivity for DRESS (>50%) than SJS/TEN.^274^ No randomized trials on treatment have been conducted. Therefore, recommendations are based on large consecutive case series and expert view (Table 2).^271^^,^^275^, ^276^, ^277^, ^278^, ^279^, ^280^ Recurrency and long-term sequelae^271^ should be assessed at 2, 3, 4, 5, 6, and 12 months and then annually.

**Table 2:** Treatment of DRESS. Ref. 271,275, 276, 277, 278, 279, 280

Drug avoidance • All potentially causal drugs should be discontinued, since early withdrawal of the culprit drug can improve prognosis. • If allergy tests are negative for a suspected drug, this should be avoided when alternatives are available. If there is no alternative after assessing the risk/benefit balance, a gradual challenge test under strict medical surveillance may be performed. • It is advisable to avoid antibiotics and nonsteroidal anti-inflammatory drugs in the acute phase that can delay the diagnosis.
Mild disease • High/very high potency topical corticosteroids 2–3 times daily • Systemic anti-H1 antihistamines for pruritus • Emollients for rash • Antipyretics • Fluid, electrolyte and nutritional support
Moderate-severe disease • Intensive care unit or burn unit admission should be considered. • Systemic glucocorticoids should be given in case of moderate-severe organ involvement, such as liver (≥5 × upper limit of normal (ULN) for alanine aminotransferase (ALT) ≥2 × ULN for alkaline phosphatase (ALP) > 5 times ULN), >3 × ULN for ALT and total bilirubin ≥2 × ULN or symptomatic hepatitis), kidney (serum creatinine ≥2.0–2.9 times baseline and urine output <0.5 mL/kg/h for ≥12 h), lungs or heart. Oral prednisone (1–2 mg/kg/day) should be given and tapered by 5–10 mg/week in 3 weeks-3 months–6 months because of the risk of relapses. Intravenous methylprednisolone at 30 mg/kg for 3 days, may be helpful in the acute phase. When patients do not respond, relapse with tapering corticosteroids or when corticosteroids are contraindicated • Cyclosporine was effective in case reports. • Intravenous immunoglobulins added to systemic corticosteroids can be considered. They were beneficial in case series of children but induced serious adverse events or persistent symptoms when given without steroids. • In patients with CMV viral load, especially with life-threatening signs of infection, antiviral drugs (ganciclovir, valganciclovir) could be considered. • In refractory patients, plasmapheresis, cyclophosphamide, azathioprine, rituximab, infliximab, anti–IL-5., anti–IL-5R and mycophenolate have been used. N-acetylcysteine can be used in DRESS by anticonvulsants since is involved in the detoxification.

### Stevens-Johnson Syndrome (SJS)/Toxic epidermal necrolysis (TEN) (Berges-Gimeno)

SJS and TEN are life-threatening skin reactions characterized by extensive epidermal necrosis of skin and mucosa. SJS is defined as less than 10% body surface area (BSA) affected by detached and detachable skin, SJS/TEN overlap 10%–30% BSA and TEN is defined as 30% or more BSA detached.^281^

SJS and TEN in children are extremely rare.^282^^,^^283^ Antiepileptic drugs and antibiotics (sulfonamides and β-lactams) are the most common groups of culprit drugs.^170^^,^^284^, ^285^, ^286^, ^287^ The main causes of SJS/TEN are drugs, but some infections have been reported as etiologic agents.^170^^,^^284^, ^285^, ^286^, ^287^ Recently, a new classification for severe blistering cutaneous reactions in children has been proposed and includes as an entity, reactive infectious mucocutaneous eruption (RIME), characterized by significant mucosal involvement with variable cutaneous involvement generally associated with respiratory infections. Mycoplasma pneumoniae is a common cause.^288^^,^^289^ Early recognition of this clinical presentation and diagnosis with nasopharyngeal polymerase chain reaction and serology is crucial to prescribe an appropriate treatment and follow-up to identify recurrence.

A precise clinical history of the patients is key to recognizing these reactions and to determine the likelihood of a drug exposure resulting in a SJS/TEN.^282^, ^283^, ^284^, ^285^ The identification of the culprit drug is often difficult, especially when multiple drugs are involved. ALDEN score is a specific algorithm of drug causality for TEN developed primary in adults but can be applied in children. An algorithm of drug causality for causality for epidermal necrolysis, ALDEN, with a score ≧ 4 considers the drug a probable cause of TEN.^290^ Lymphocyte transformation test (LTT) can be helpful for drug causality evaluation in pediatric populations who developed SJS/TEN, although further research in is need to standardized and evaluate LTT in children.^84^^,^^90^^,^^291^ Drug patch tests have low sensitivity for SJS/TEN, and pediatric data remain scarce.^56^ Drug provocation testing with suspected drug is contraindicated in SJS/TEN.^64^ Skin biopsy maybe considered if diagnosis is unclear and to exclude other blistering disease but not necessary for diagnosis.^292^

Survival in children with SJS and TEN is significantly better than that reported in adults.^282^^,^^283^ Nevertheless, pediatric SJS/TEN has high rates of complications, sequelae, and recurrences. Follow-up by multidisciplinary team (allergology, dermatology, ophthalmology, infectious diseases, gynecology, urology and psychology/psychiatry) ideally with experience in SJS/TEN may be required. The SCORTEN (severity-of-illness score for TEN) is a disease severity score specific for TEN, based on 7 clinical and laboratory factors. It is a useful predictor of morbidity in adult and can predict morbidity in pediatric patients.^293^ Children with a high initial SCORTEN, and a history of malignancy and stem cell transplant are considered high-risk patients.^294^

The early recognition of SJS/TEN and withdrawal of potential causative drugs is crucial to prevent morbidity and mortality. The therapeutic strategies of managing pediatrics SJS/TEN include supportive care to maintain hemodynamic stability and preventing life-threatening complications, and immunomodulatory treatments. Systemic corticosteroids and intravenous immunoglobulin appear of any benefit only if introduced extremely early.^295^ Recent studies show that TNF-α inhibitors (infliximab and etanercept) could be as an effective alternative.^296^ To date there is little evidence to support which is the best immunomodulatory treatment. The decision to administer immunosuppressive treatment should be made by experienced multidisciplinary teams and in a personalized manner for each patient.^297^ It is vital that high risk patients, including those with extensive skin loss, systemic involvement or comorbidities, receive treatment in a pediatric intensive care setting and by a team specialist expertise.^297^

## Raising awareness on a newly identified type of reaction

### Drug-induced enterocolitis syndrome in children (DIES) (Attanasi)

DIES is defined as a rare non-IgE-mediated allergy syndrome with the same clinical characteristics as food protein induced enterocolitis syndrome (FPIES).^298^^,^^299^ In an Italian pediatric allergy unit, the incidence was estimated to be equal to 0.4% in the period from 2014 to 2019, although it is still to be defined.^300^ To date, 12 case reports of DIES were described in the literature: 9 in children^1^^,^^3^, ^4^, ^5^, ^6^, ^7^, ^8^ and 3 in adults.^306^, ^307^, ^308^ The causative drugs were amoxicillin or amoxicillin/clavulanate in 10 cases,^288^^,^^300^, ^301^, ^302^, ^303^^,^^305^^,^^307^^,^^308^ pantoprazole in an adult,^306^ and paracetamol in an infant.^304^ In children with DIES the mean age at the onset of the symptoms is 6.3 years and ranges from 10 months to 14 years.^298^^,^^300^, ^301^, ^302^, ^303^, ^304^, ^305^ In 2019,^302^ the diagnostic criteria for patients with possible DIES based on FPIES was propused^309^ (Table 3). The differential diagnosis of vomiting occurring after a drug administration includes also IgE-mediated drug hypersensitivity reactions (IgE DHRs) and drug side effects. Importantly, the latency period, the clinical picture and the immunological mechanisms differentiate DIES from IgE DHRs and drug side effects.^299^^,^^300^^,^^302^ Indeed, the latency period is longer >1.5–2 h in DIES than in IgE DHRs (ie, vomiting typically appears within minutes or by no later than 1 h) and vomiting is protracted and associated with poor general condition. Drug side effects arise from their pharmacological actions rather than immunological mechanisms.^310^ These side effects are generally more common and well-documented for each specific drug, and factors such as dose, frequency and route of drug can influence their occurrence.^310^ From the pathological point, the release of toxic or immunogenic metabolites after the first-pass hepatic and intestinal processing of the drug might cause the drug-induced intestinal damage.^311^ In DIES, patients showed a good response to anti-emetics (ondansetron) and intravenous fluids and corticosteroids, as opposed to adrenaline, which was found to be ineffective.^302^^,^^308^

**Table 3:** Clinical presentation, diagnostic criteria and treatment in children and adolescents with drug-induced enterocolitis syndrome (DIES).

	DIES characteristics	
**Clinical presentation** Ref. 299,301	Repeated delayed vomiting, diarrhea and abdominal pain (less frequent), pallor, hypotension and tachycardia which may progress to dehydration and hypovolemic shock.	
a**Diagnostic criteria** Ref. 302	***Major criteria*** Vomiting in the 1- to 4-h period after ingestion of the causative drug and absence of classic IgE-mediated allergic skin or respiratory symptoms.	***Minor criteria*** 1. A second episode of repetitive vomiting after ingestion of the same drug; 2. Repetitive vomiting episode 1–4 h after ingestion of a different drug 3. Extreme lethargy 4. Marked pallor 5. Need for ED 6. Need for IV fluids support 7. Diarrhea in 24 h (usually 5–10 h) after drug intake 8. Hypotension 9. Hypothermia
**Pathological mechanisms** Ref. 311, 312, 313, 314, 315, 316	The release of toxic or immunogenic metabolites after first-pass hepatic and intestinal processing of the drug might cause the drug-induced intestinal damage. It is considered a cell-mediated disorder characterized by: 1. Activation of immune cells, such as mast cells, neutrophils and T cells. 2. Release of pro-inflammatory cytokines: • ↑ IL-8 • ↑ bIL-9 • ↑ bTNF-α • ↓ TGF-β • ↑ bINF-γ • ↑ histamine Additionally, metabolomics approach revealed that the activation of the purinergic pathway might be a possible pathological mechanism linked with the inflammation and vomiting, triggering the serotonin release from gastric and duodenal mucosa.	
**Laboratory exams** Ref. 298,301,304,305,307	- Neutrophilic leukocytosis and an increased level of cmethemoglobin. - Tryptase dosage was always normal. - The only biomarker described in the literature was ECP which was increased in the stool samples from 24 to 48 h after a DPT with AMX/CLV.	
**Allergy tests** Ref. 298,300,302,304,305,317,318	- Serum drug specific IgE, SPT and IAT were always negative. - APT for AMX was evaluated in a child with DIES and resulted negative. However, positive ATP for drugs varies widely in literature, especially among children (0.9–89%).	
**Acute phase therapy** Ref. 302,308	dOndansetron, systemic corticosteroids and IV fluids. Epinephrine is found to be ineffective.	
**Differential diagnosis** Ref. 300,310	The latency period.the clinical picture and the immunological mechanisms differentiate DIES from IgE DHRs and drug side effects. In IgE DHRs the vomiting typically appears within minutes or by no later than 1 h after drug intake and is commonly associated with involvement of another organ system due to histamine liberation, such as skin rash or swelling. Drug side-effects are not based on immunological mechanisms but on its pharmacological effects and are more common and usually well recognized for each particular drug. Additionally, dose, frequency and route of drug might be helpful to differentiate allergic reactions from side effects or intolerance.	
**Cross-reactivity** Ref. 300	To date no studies evaluating cross-reactivity among drugs.	
**Unanswered questions** Ref. 300	Currently not investigated are: 1. Natural history 2. Possible presence of predisposing genetic factors 3. Potential existence of atypical or chronic forms	

IgE: immunoglobulin E; ED: Emergency Department; IV: intravenous; IL: interleukin; TNF: tumor necrosis factor; INF: interferon; TGF: transforming growth factor; ECP: eosinophil cationic protein; AMX/CLV: amoxicillin/clavulanate; SPT: skin prick test; IAT: intradermal allergy testing; ATP: atopy patch test; IgE DHRs: IgE-mediated drug hypersensitivity reactions.

a The diagnosis of DIES requires that a patient meets the major criterion and >3 minor criteria and is occasionally confirmed by a drug provocation test (DPT). Importantly, a diagnostic DPT should be strongly considered to confirm the diagnosis if only a single episode of vomiting has occurred. No consensus has been reached regarding the starting dose for the oral provocation test in either FPIES or DIES.

b Peripheral antigen-specific T cell-derived pro-inflammatory cytokines known to regulate the intestinal barrier permeability.

c Methemoglobinemia is caused by the increased oxidation of iron in hemoglobin due to nitrites and nitrates released during inflammation.

d Ondansetron, a serotonin 5-HT3 receptor antagonist, is reported as a successful treatment for vomiting, abdominal pain, and lethargy and is contraindicated in infants <6 months of age and in children with cardiac disease as it might cause QT prolongation.

## Multidisciplinary programs to address highly complex drug allergy

### Hypersensitivity to chemotherapy and biologics (Madrigal-Burgaleta and Alvarez-Cuesta)

Every year, approximately 400,000 children and adolescents worldwide are diagnosed with various cancers, including leukemia, brain cancer, lymphoma, and solid tumors.^319^^,^^320^ Chemotherapy remains pivotal in cancer treatment, while biologics find broader applications in inflammatory and autoimmune conditions.^321^ Unfortunately, the incidence of adverse reactions to these drugs is rising.^321^^,^^322^

DHRs to chemotherapy and biologic therapeutics in children can be severe, leading to treatment discontinuation, threatening their quality of life and survival.^15^^,^^323^ Commonly implicated drugs include platinum compounds, methotrexate, l-asparaginase, and monoclonal antibodies.^321^^,^^323^

Despite the challenges these medications pose, allergy-led initiatives addressing DHRs to chemotherapy and biologics have yielded excellent results.^65^^,^^324^, ^325^, ^326^, ^327^ WAO advocates for allergists to spearhead multidisciplinary teams, ensuring swift access to high-quality care for these patients.^15^ Recent guidance offers strategies for establishing drug desensitization hubs, including supplemental material with a practical model for positively transforming allergy care across all age groups.^15^ (see also Supplemental Appendix 1 for an adaptation to pediatric allergy centers).

While general principles guide DHR assessment and management in children and adults, specific adjustments are necessary.^328^ Although crucial for phenotyping and risk assessment, clinical history poorly predicts hypersensitivity to chemotherapy and biologics.^325^^,^^329^^,^^330^ Moreover, clinical history in children has some caveats that make it more unreliable, necessitating confirmatory testing, primarily through skin testing and drug challenge, owing to the limited availability of *in vitro* options.^15^^,^^255^^,^^331^, ^332^, ^333^

When performed in dedicated spaces and by experts in drug allergy, rapid drug desensitization (RDD) cost-effectively allows allergic patients to safely receive their first-choice treatments and maintain the same survival outcomes as their non-allergic counterparts.^15^^,^^65^^,^^324^^,^^326^^,^^328^

**Fig. 1:**
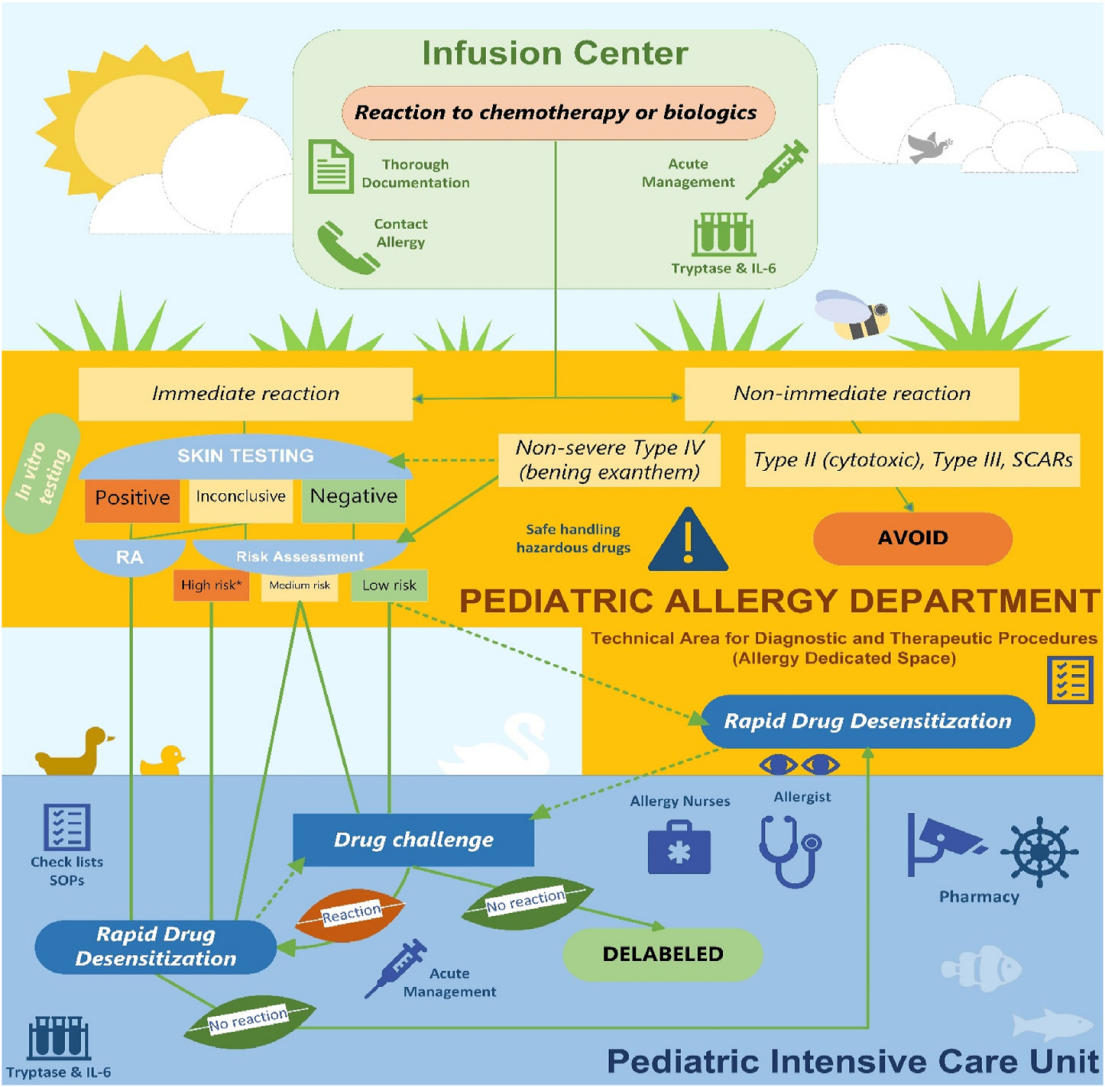
Algorithmic Approach to Pediatric Drug Hypersensitivity Reactions (DHRs) to Chemotherapy and Biologics. ∗High-risk: The definition of high-risk should be local and incorporate factors beyond the severity of the index reaction, as per WAO guidance.^15^ A recent publication is an excellent example of how to apply risk assessment locally.^327^ Note on intensive care: As groups gain experience or expand capacity, they may eventually transition all procedures from intensive or semi-intensive care to an allergy-led technical area.^326^ Local adaptation is essential, including considerations for safe handling of hazardous drugs.^65^ A dotted line represents “selected cases”; IL-6, interleukin 6; RA, risk assessment; SCARs, severe cutaneous adverse reactions, such as drug reaction with eosinophilia and systemic symptoms (DRESS), Stevens-Johnson's Syndrome (SJS), or toxic epidermal necrolysis (TEN); SOPs, standard operating procedures

Fig. 1 illustrates the diagnostic and therapeutic framework for managing pediatric DHRs to chemotherapy and biologics. Supplemental Table 10 delineates nuances and practical considerations based on previous experience.^15^^,^^65^^,^^321^^,^^328^^,^^334^^,^^335^ Supplemental Table 11 shows unresolved challenges. Supplemental Appendix 1 provides guidance on how an allergy-led technical area can be organized to incorporate complex care.

### Drug hypersensitivity reactions (DHRs) in cystic fibrosis (CF) (Madrigal-Burgaleta and Alvarez-Cuesta)

DHRs in CF patients pose significant challenges due to potential limitations in antibiotic options.^336^ However, studies suggest that true drug allergies might be comparable to the general population.^337^, ^338^, ^339^, ^340^ Most DHRs are delayed-type reactions rather than immediate, most being mild cutaneous manifestations.^341^^,^^342^ While anaphylaxis remains rare, severe reactions can occur.^338^^,^^343^

The primary causative agents include beta-lactams, particularly ceftazidime and piperacillin-tazobactam, critical antipseudomonal agents.^344^ The emergence of cystic fibrosis transmembrane conductance regulator (CFTR) modulator drugs has revolutionized CF care, but reactions to these medications have been reported.^343^^,^^344^

Many reactions are not true allergies but non-specific exanthems or other phenomena.^15^^,^^345^ “Treating-through” exanthems can help when the risk assessment is favorable.^344^^,^^346^ Inaccurate diagnosis still leads to unnecessary avoidance of effective therapies, increasing the risk of using less effective, more toxic, or costly alternatives, which may compromise patient outcomes.^335^^,^^336^

Diagnosing DHRs has some limitations. I*n vitro* tests are promising but not widely available and poorly validated in pediatric patients.^48^^,^^210^^,^^255^^,^^343^ Skin testing and drug challenges require caution and expertise due to their technical complexity and the risk of severe reactions.^15^^,^^48^^,^^210^ Pediatric patients might be prone to false positives during challenges, which might need repeating.^335^

RDD can help both in immediate reactions and selected milder non-immediate reactions.^328^^,^^329^^,^^347^^,^^348^ However, publications without confirmatory allergy workup, including ST and drug provocation test (DPT), should be viewed critically.^15^

**Fig. 2:**
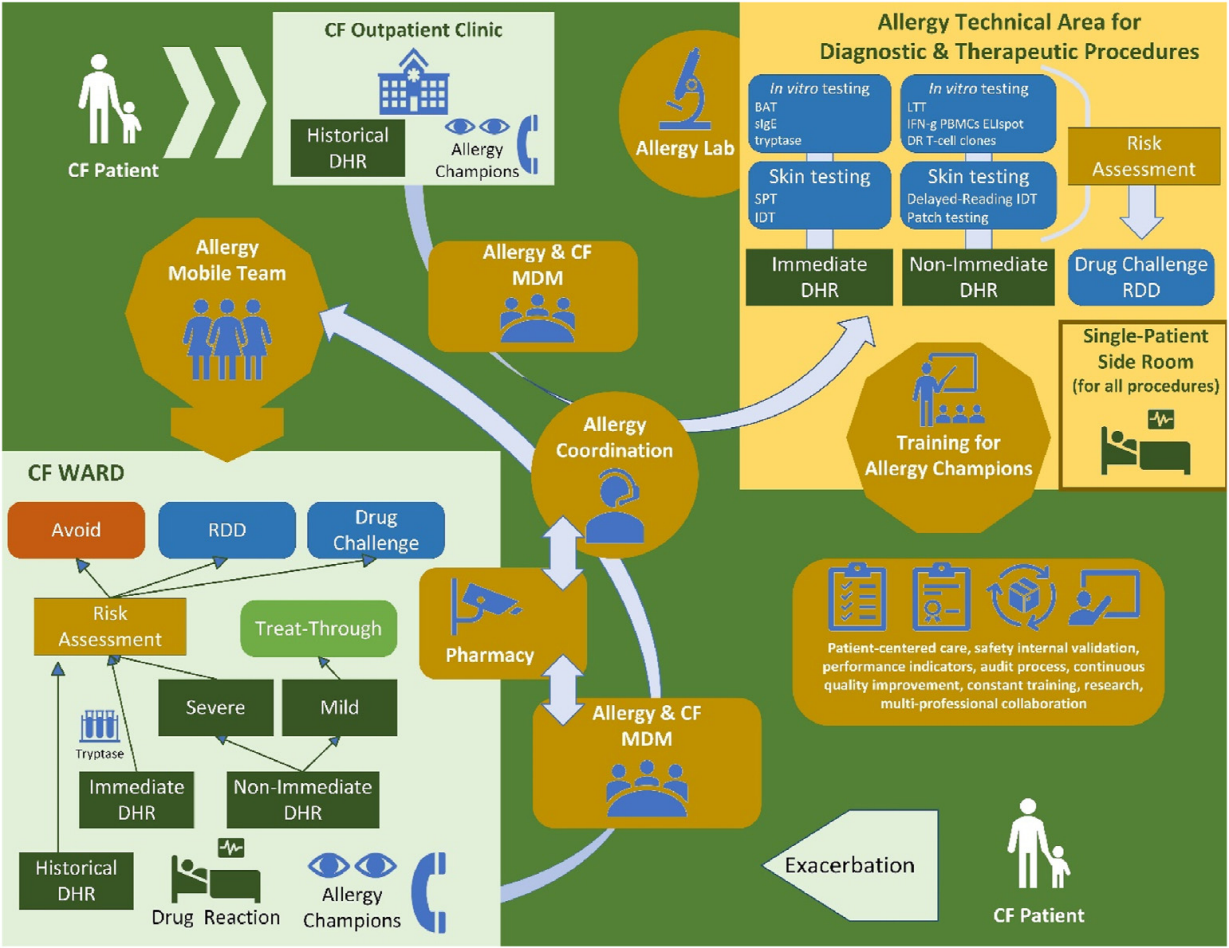
Integrated Allergy Care Pathways for Pediatric Cystic Fibrosis Patients: A Comprehensive Approach to Patient-Centered Management. Note: Allergy champions are healthcare professionals who, regardless of their role or speciality, actively engage in coordinating and promoting allergy care within their respective areas. They serve as points of contact and liaise with the allergy team to ensure effective, safe, and patient-centered management. CF, cystic fibrosis; DHR, drug hypersensitivity reaction; DR T-cell clones, drug-responsive T-cell clones; IDT, intradermal testing; MDM, multidisciplinary meeting; RDD, rapid drug desensitization; SPT, skin prick testing.

Multidisciplinary collaboration is pivotal in managing DHRs in CF patients, underscoring the crucial role of allergists.^15^^,^^336^ Standardized diagnostic algorithms and rapid access to testing are essential.^334^^,^^344^ Infection control measures can be challenging for allergists, as these patients should avoid non-essential face-to-face consultations and undergo testing in the CF ward (with a “mobile allergy team”) or in single-patient side rooms of the allergy-led dedicated area (See Supplemental Appendix 1 for an example of an allergy-led technical area integrating complex care).^15^^,^^349^ See Fig. 2.

### Peri-operative drug allergy (POH) (Powell and du Toit)

POH occurs at a lower incidence than in adults^350^ (1:2100^351^ to 1:36479 anaesthetics).^352^ Investigating POH in children is complex given the difficulty of skin testing young children and the unknown sensitivity and specificity at this age.^353^ Accurate identification of culprit medications is the most important factor to reduce risk for future surgery.^351^^,^^354^^,^^355^

Features of POH include severe hypotension (often the presenting finding),^356^^,^^357^ bronchospasm,^358^ and tachycardia. Bradycardia can also occur.^356^ Milder reactions are characterised by cutaneous features and angioedema.^359^ Cutaneous features are infrequent with severe reactions.^356^^,^^357^ Seeking anaesthetic opinion can help differentiate POH from known adverse anaesthetic drug reactions and possible effects of the surgical procedure and complications thereof.^360^

IgE and non-IgE mediated POH can present with similar findings.^350^^,^^356^ A proven IgE cause is found after testing in 28%^355^ to 75%^351^ of cases. Earlier studies identified latex as the main cause of POH^361^ but this is now a rare cause. Recent studies identify antibiotics and NMBA as commoner culprits^356^^,^^357^ (Supplemental Table 12).

Principles of investigation in children and adults are similar^353^ but intradermal testing is painful, technically demanding, and should be directed to the most likely culprits. Communication regarding anaesthetic needs for future planned procedures helps direct investigations. A multi-disciplinary team should facilitate testing (Supplemental Fig. 10) and consider the clinical correlation with test results.^353^^,^^362^, ^363^ For medications where there is a disparity between results and clinical presentation drug challenges may be required.^353^ This may also be needed for medications, such as NSAIDs, where skin testing may be unhelpful. Where test results are negative, but there is clinical suspicion of a causative agent, this suspected trigger should be avoided, and a safe alternative identified.^353^ Clear advisory plan and documentation for the future is required (Supplemental Table 13).

## CONCLUSION (Berges-Gimeno)

Children and adolescents have a low incidence of DHRs, although they can present potentially severe reactions. Improving communication channels and establishing close collaboration with primary care physicians and other specialists to quickly refer children who have a history of DHR to the allergy department is essential to avoid unnecessary medication restrictions that may persist into adulthood. A proper and personalized allergy diagnostic approach is crucial to prevent mislabeling and to achieve a true diagnostic.

This WAO Statement updates the management of DHRs in children and seeks to provide the best and safe strategies for framing main drugs, potentially severe reactions and newly emerged reactions like DIES. The design of an allergy study of DHRs should be based on critical aspects such as risk stratification, clinical manifestation, the culprit drug, suspected mechanism of the reaction and biomarkers avalaible.The allergy workup and management of DHRs in children and adolescents should be performed in specialized centers by experienced allergists.

## Availability of data and material

Not applicable.

## Author contributions

Berges-Gimeno (Lead of the document's Task Force), Ansotegui (Executive Medical Director of WAO), Alvarez-Cuesta (Co-Chair of the WAO Drug Hypersensitivity Reactions Committee), and Madrigal-Burgaleta (Co-Chair of the WAO Drug Hypersensitivity Reactions Committee) contributed to conceiving, designing, editing, and revising the manuscript.

Steering Committee Authors Berges-Gimeno, Alvarez-Cuesta, Atanaskovic-Markovic, Attanassi, Caffareli, Caubet, du Toit, Guzman-Melendez, Kuyucu, Madrigal-Burgaleta, Mayorga, Powel, Ramien, Rebelo Gomes, Mori, and White, performed the literature research and drafted their specific sections.

Berges-Gimeno and Guzman-Melendez co-authored the Introduction section. Berges-Gimeno and Mayorga co-authored the Allergy Diagnostic Workup section. Atanaskovic-Markovic, Mori, White, Caubet, Kuyucu, and Rebelo Gomes co-authored the Evaluation and Management of Specific Drugs section. Caffareli and Ramien co-authored the Severe Cutaneous Adverse Reactions section. Attanasi authored the Raising Awareness on a Newly Identified Type of Reaction section. Madrigal-Burgaleta, Alvarez-Cuesta, du Toit, and Powell co-authored Multidisciplinary Programs to Address Highly Complex Drug Allergy section. Berges-Gimeno authored the Conclusion section. All the Steering Committee authors accepted the final version of the manuscript. Any supplementary material was kindly provided individually by the authors of each section. A Review Panel of expert allergists from all around the globe critically reviewed the manuscript. All the review panel members sent a review with modifications to the manuscript that were duly implemented and equally contributed to the discussions.

## Ethics approval

Ethics approval not applicable.

## Consent for publication

All authors gave their consent to publish this work.

## Submission Declaration

The authors confirm that this manuscript is original, has not been published before, is not currently being considered for publication elsewhere.

## Funding

Not applicable.

## Declaration of competing interest

The authors report no competing interests with regards to this document.
