# Supplementary material for: Drug hypersensitivity reactions in children in clinical practice: A WAO Statement
Source: World Allergy Organ J. 2025 Aug 29;18(9):101087. doi: 10.1016/j.waojou.2025.101087 (PMC12419032; doi:10.1016/j.waojou.2025.101087)
Supplement: Multimedia component 1 [file mmc1.docx]

**SUPPLEMENTAL TABLE 1: Maturational changes that affect pharmacokinetics in childhood**^(16, 17, 18, 19)^

| Absorption | Distribution | Metabolism | Excretion |
| --- | --- | --- | --- |
| Smaller length of GI tract  Poor anti reflux mechanism in the firsts 3 months  Minor production of gastric acid before 3 years  Gastric emptying delayed in neonates and infants  Transit time shorter in young children  Minor secretion and activity of bile and pancreatic fluid in neonates and infants  > transdermal absorption in neonates and infants  Variable absorption after intramuscular administration  Rectal route not modified by age (it depends on formulation: solutions vs suppositories)  Different architecture of lungs and ventilatory capacity for inhaled drugs in infancy | Higher membrane permeability in immature neonates, including BBB  Continue myelinization pre and post birth  Disproportional larger brain in young children  Plasma protein content decreased in neonates and infants  Reduced protein binding and unbound fraction of drugs higher in pediatric population  Albumin affinity of many drugs decreased in neonates  More content of water in neonates and infants | Disproportional larger liver in neonates and infants  Immature enzymes, especially microsomal, in neonates  Decreased activity of Phase I enzymes (oxidative, reductive, hydrolytic and epoxide-hydrolases) in pediatric age  Decreased activity of Phase II enzymes, involved in important drugs conjugation process, in pediatric age  Immature renal drug metabolism in neonates  Bacterial drug metabolism affected by route of delivery, diet, and early antibiotic prescription | Immature renal function in infants before 3 months, affecting glomerular filtration, tubular excretion and tubular reabsorption.  Minor drug biliary excretion in neonates  Limited knowledge about salivary drug excretion in neonates and acutely ill children |

BBB: Blood-brain barrier

**SUPPLEMENTAL TABLE 2: Specific aspects on *in vitro* DHRs evaluation in children**

| **Specific aspects on *in vitro* DHRs evaluation in children** |
| --- |
| - Same rules as adults should be applicable. - Sample volume limitations sometimes needed for particular procedures. - Differences in the immunologic responses having altered capacity to generate Th1/Th2 responses. - Interaction with underlying pathologies like atopy and infection diseases concomitant with drug administration. - Children present, in general, less complex drug-related allergies than adults because they do not tend to take many drugs at the same time. |

**SUPPLEMENTAL TABLE 3. In vitro diagnostic Test for drug hypersensitivity reactions in children**

| **Methods** | **Drugs** | **N. Patients** | **Sensitivity**  **(%)** | **Specificity (%)** | **NPV**  **(%)** | **PPV (%)** | **Ref** |
| --- | --- | --- | --- | --- | --- | --- | --- |
| Immunoassay | BLs (PENI) | 732 children | 2.9% | 99% | --- | --- | 67 |
| BAT | ATRAC | 75 | 63% | 100% | 70% | 100% | 69 |
| BAT | ROC* | 272 | 92% | 93% | ----- | ------ | 70 |
| BAT | NMBA | 120 | 77% | 76% | ----- | ------ | 71 |
| BAT | AX or AX-CLV | 18 children & 21 adults | 38.5% | 100% | ---- | ---- | 72 |
| BAT | BLs | 14 (IDHR and NIDHR) | 50% | 91.5% | ---- | ---- | 74 |
| Tryptase | Perioperatives | 82 | 78% | 91% | 98% | 44% | 76 |
| Histamine | CLV | 39 | 55% | 85% | 76% | 69% | 77 |
| Tryptase | BLs | NN | 30 - 94.1% | 92.3 - 94.4% | ---- | ---- | 77 |
| LTT | PHE, PHENY, CARBA; LAMO | 24 | 58.4% | 95.8%, | 93.3% | 69.9% | 79,80,82 |
| LTT | PHENY | 7 | 100% | 90.9% | 87.5% | 100% | 87 |
| LTT | AX | 9 patients & 16 controls | 60–70% | 85 - 99%. | ---- | ---- | 74,88 |
| LTT | AX or AX-CLV | 50 or 194 children | 52 - 63.6% | 92 - 100% | 86 -100% | 60 -65% | 83,64 |
| LTT | CLV | 17 | 33.3% | 100% | 60% | 100% | 64 |
| LTT | AX or AX-CLV | 10 | 40-70% | 100% | ---- | ---- | 86,89 |
| LTT | Multiple drugs | 16 children Acute/Postrecovery phases | 77.8/33.3% | ---- | ---- | ---- | 84 |
| LTT | Multiple drugs | 9 Children/31Adults Acute/Postrecovery phases | 50/25% | 95.11 | ---- | ---- | 85 |
| ELISpot (IFN-γ) | Multiple drugs | 16 children Acute/Postrecovery phases | 88.9/66.7% | ----- | ---- | ---- | 84 |
| ELISpot (IL-4) |  |  | 100/66.7% | ----- | ---- | ---- |  |
| ELISpot (IFN-γ/IL-4) |  |  | 100/83.3% | ----- | ---- | ---- |  |
| ELISpot (IFN-γ) | Multiple drugs | 9 Children/31Adults Acute/Postrecovery phases | 77/50% | 82.85 | ---- | ---- | 90 |
| ELISpot (IL-4) |  |  | 67/50% | 92 | ---- | ---- |  |
| ELISpot (IFN-γ/IL-4) |  |  | 82/67% | 77.27 | ---- | ---- |  |
| ELISpot (IFN-γ) | PHENY | 7 | 71.4% | 100%100% | 100% | 84.6% | 87 |

* Although several NMBAs are analysed, data are provided for only one of them; PENI: Penicillins; BLs: Betalactams; PENI: Penicillins; ATRAC: Atracurium; ROC: Rocuronium; NMBA: Neuromuscular blocking agents; AX: Amoxicillin; AX-CLV: Amoxicillin-Clavulanic acid; PHE: Phenobarbital; PHENY: Phenytoin; CARBA: Carbamazepine; LAMO: Lamotrigine; IDHR: Immediate drug hypersensitivity reactions; NIDHR: Non-immediate drug hypersensitivity reactions; BAT: Basophil activation test; LTT: Lymphocyte transformation test; ELISpot: Enzyme-Linked ImmunoSpot Assay; NN: Not noted.

**SUPPLEMENTAL TABLE 4: In vitro testing for drug hypersensitivity reactions**

| **Methods** | **Phenotype** | **Endotype** | **Limitation** | **Improvement** |
| --- | --- | --- | --- | --- |
| Immunoassay | IDHRs | Tryptase or histamine | - Do not identify the responsible drug. - It must be measured at onset (30-120 minutes). - Levels should be compared to baseline levels. - Concomitant mast cells disorders could increase basal and acute tryptase levels. | The formula: [1.2 × (Basal tryptase value) + 2 μg/L] |
| Immunoassay | IDHRs | Drug-sIgE | - Limited to a low number of drugs. - Low sensitivity. - False positive results for PV. | - Use different drug metabolites. - Increase of drug density in solid phase. |
| BAT | IDHRs | Basophil activation upon the suspected drug stimulation | - Low sensitivity. - Not useful for evaluating non-immunological reactions like NSAIDs hypersensitivity or MRGPRX2 mediated reactions. - Non responders produce not valid results | - Use of different activation markers. - Use different drug metabolites. |
| LTT | NIDHRs | Lymphocyte proliferation upon the suspected drug stimulation. | - Complexity of the procedure. - Need for qualified laboratories and personnel. - LTT based on the detection of radiation. | - Use of professional antigen presenting cells. - Evaluation in effector cells. - Assessing cells proliferation with CFSE (carboxyfluorescein diacetate succinimidyl ester) or BrdU (bromodeoxyuridine) by flow cytometry. |
| ELISpot | NIDHRs | Nº cells releasing cytokine or cytotoxic markers | - Complexity of the procedure. - Need for qualified laboratories and personnel. | - Use of professional antigen presenting cells. - Evaluation in effector cells. |

BAT: Basophil activation test; LTT: Lymphocyte transformation test; ELISpot: Enzyme-Linked ImmunoSpot Assay; IDHR: Immediate drug hypersensitivity reactions; NIDHR: Non-immediate drug hypersensitivity reactions; PV: Penicillin V; NSAIDs: Non-steroidal anti-inflammatory drugs; MRGPRX2: MAS related GPR family member X2; CFSE: carboxyfluorescein diacetate succinimidyl ester); BrdU: Bromodeoxyuridine.

**SUPPLEMENTAL FIGURE 1**. Algorithm for the diagnosis of immediate hypersensitivity reactions to beta-lactams

+

-

+

Drug avoidance

-

+

Confirmed drug hypersensitivity

Skin tests (prick, IDTs)

Drug provocation test

Mild reactions

Urticaria, Angioedema

sIgE

Clinical history of possible immediate hypersensitivity reactions to beta-lactams

Severe reactions

Anaphylaxis

--

No drug avoidance

Delabel allergy

**SUPPLEMENTAL TABLE 5**. Highest nonirritating concentrations recommended for both prick and intradermal testing with beta-lactams ^(94)^

| Hapten | Concentration |
| --- | --- |
| Benzylpenicilloyl octa-L-lysine (BP-OL)  Benzylpenicilloyl poly-L-lysine (PPL) | 0.04 mg/ml |
| Benzylpenicillin and sodium benzylpenilloate (MD) | 0,5 mg/ml |
| Benzylpenicillin | 10 000 IU/ml |
| Amoxicillin and other semi-synthetic penicillins | 20-25 mg/ml |
| Clavulanic acid | 20 mg/ml |
| Cefepime | 2 mg/ml |
| Other cephalosporins | 20 mg/ml |
| Aztreonam | 2 mg/ml |
| Imipenem-cilastatin | 0,5 mg/ml |
| Meropenem | 1mg/ml |

**SUPPLEMENTAL FIGURE 2**. Algorithm for the diagnosis of non-immediate hypersensitivity reactions to beta-lactams

Drug avoidance

Confirmed drug hypersensitivity

Mild reactions

MPE, SSLR

Severe reactions

SCARs

Clinical history of possible non-immediate hypersensitivity reactions to beta-lactams

*In vitro* test

(LTT ili IFNγ ELISpot)

-

Drug provocation test

Skin tests (patch, IDTs)

-

+

-

+

No drug avoidance

Delabel allergy

No drug avoidance

Delabel allergy

**SUPPLEMENTAL TABLE 4:** Non- Betalactams Antibiotics Skin Tests concentrations^1,2,3,4,5,6^

| Non-betalactam antibiotics | Name | skin prick test concentrations | intradermal test concentrations | Patch test  concentrations |
| --- | --- | --- | --- | --- |
| Sulfonamides | Cotrimoxazole | 80 mg/ml | 0.8 mg/ml | 5-30 % dilution (pet),  80 mg/mL dilution (aq), 10-50 % dilution (DMSO: dimethyl sulfoxide) |
| Macrolides | Clarithromycin | 50-0.1-10 mg/ml | 0.0005-0.005-0.05-5-0.001-0.1-1-10 mg/ml |  |
|  | Erythromycin | 10-50 mg/ml | 0.01-0.05- 0.1-1-10 mg/ml | 1-30 % dilution (pet)  Pure in powder |
|  | Azithromycin | 10-100 mg/ml | 0.01-0.1-1-10 mg/ml |  |
| Antituberculosis  drug | Ethambutol |  |  | Up to 50% dilution  (aq or pet) |
|  | Rifampicin | 60 mg/ml | 0.002-0.006-0.06-0.6-6 mg/ml | Up to 50% dilution  (aq or pet) |
|  | Isoniazid | 100 mg/ml | 10 mg/ml | Up to 50% dilution  (aq or pet) |
| Quinolones | Ciprofloxacin | 2-0.02-0.2 mg/ml | 0.02-0.025-0.005-0.006 mg/ml | 10-25% dilution (pet) |
|  | Moxifloxacin | 1.6 mg/ml | 0.025-0.005 mg/ml | 10-25% dilution (pet) |
|  | Levofloxacin | 5-0.05-0.5 mg/ml | 0.025-0.05-0.005 mg/ml | 10-25% dilution (pet) |
| Glycopeptides | Vancomycin | 50 mg/ml | 0.005 mg/ml | 10% dilution (aq) |
| Aminoglicosides | Streptomycin |  |  | 1% dilution (pet) |
|  | Gentamycin | 40 mg/ml | 4 mg/ml | 20% dilution (pet) |
|  | Tobramycin | 40 mg/ml | 4 mg/ml | 20% dilution (pet) |
|  | Clindamycin | 150 mg/ml | 15 mg/ml | 2-10-30% (pet) |
| Tetracyclines | Doxycycline | 10 mg/ml | 0.0001-0.001 mg/ml |  |
|  | Minocycline | 0.2 mg/ml | 0.0002-0.002 mg/ml |  |
|  | Tetracycline | 0.5-25 mg/ml |  |  |
|  | Tigecycline | 1 mg/ml | 0.01-0.1 mg/ml |  |
|  | Metronidazole | 125 mg/ml | - |  |

References

1. Grinlington L, Choo S, Cranswick N, Gwee A. Non-β-Lactam Antibiotic Hypersensitivity Reactions. *Pediatrics*. 2020 Jan;145(1):e20192256. doi:10.1542/peds.2019-2256.
2. Merk HF, Bickers DR. Hypersensitivity to non-β-lactam antibiotics. Allergol Select. 2022; 6: 11-17. DOI 10.5414/ALX02311E
3. Kuyucu S, Mori F, Atanaskovic-Markovic M, Caubet JC, Terreehorst I, Gomes E, Brockow K; Pediatric Task Force of EAACI Drug Allergy Interest Group Hypersensitivity reactions to non-betalactam antibiotics in children: an extensive review. Pediatr Allergy Immunol. 2014;25(6):534-543. doi: 10.1111/pai.12273.
4. Zhu LJ, Liu AY, Wong PH, Arroyo AC. Road Less Traveled: Drug Hypersensitivity to Fluoroquinolones, Vancomycin, Tetracyclines, and Macrolides.Clin Rev Allergy Immunol. 2022;62(3):505-518. doi: 10.1007/s12016-021-08919-5
5. Dilley M, Geng B. Immediate and Delayed Hypersensitivity Reactions to Antibiotics: Aminoglycosides, Clindamycin, Linezolid, and Metronidazole. Clin Rev Allergy Immunol. 2022;62(3):463-475. doi: 10.1007/s12016-021-08878-x.
6. Süleyman A, Yücel E, Tamay ZÜ, Güler N. Evaluation of Suspected

**SUPPLEMENTAL FIGURE 3:** This figure illustrates a theoretical protocol for evaluation of a pediatric patient with a history of NSAID hypersensitivity.

**Consider deferring a DPT in a child with active chronic spontaneous urticaria and a suspicion for NECD until underlying urticaria is controlled.*

DPT – drug provocation test

NECD – NSAID-exacerbated cutaneous disease

N-ERD – NSAID-exacerbated respiratory disease

NIUAA – NSAID-induced urticaria, angioedema, anaphylaxis


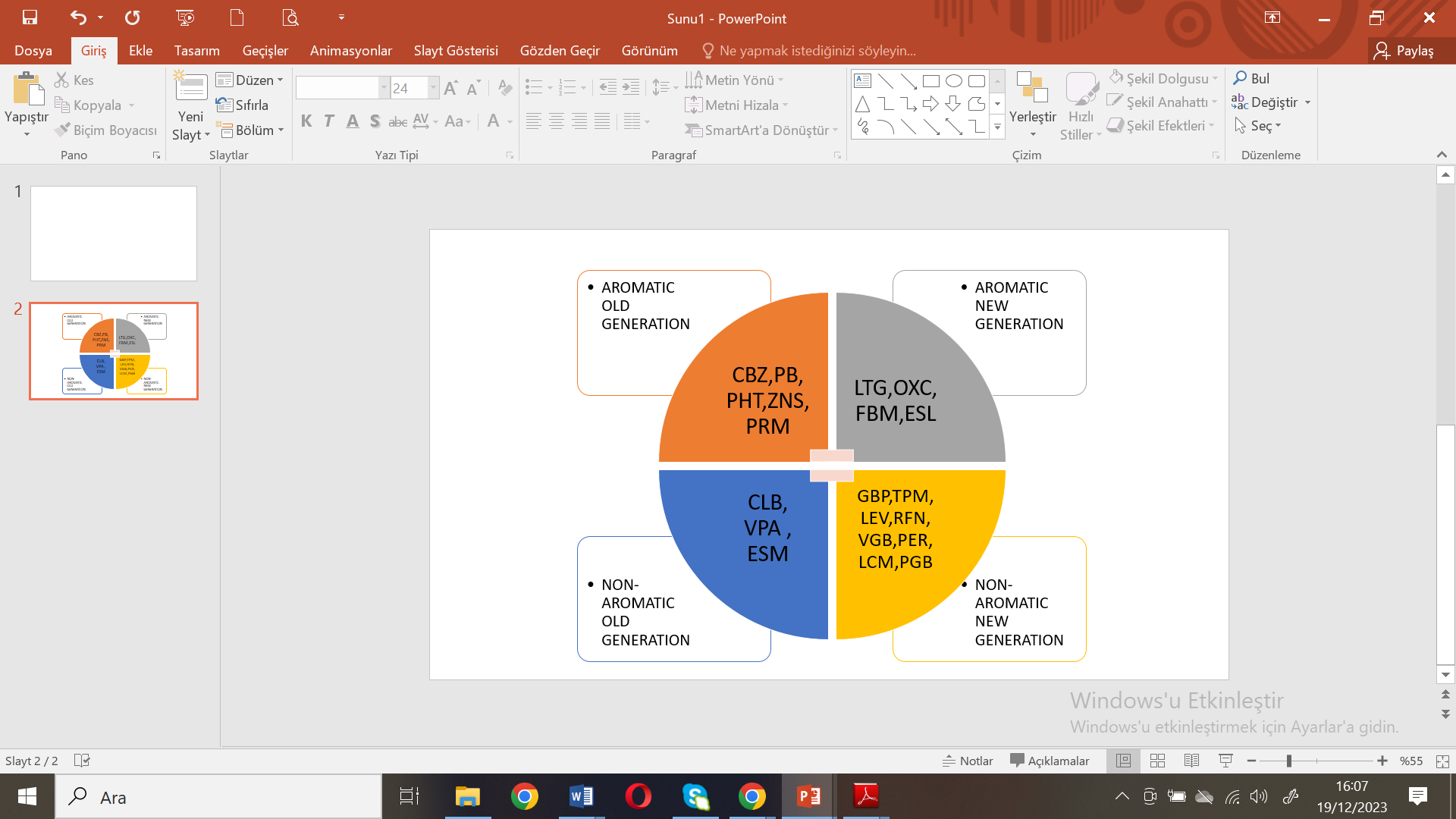


**SUPPLEMENTAL FIGURE 4** Classification of antiepileptic drugs. Formal abbreviations: carbamazepine (CBZ), phenytoin (PHT), phenobarbital (PB),zonisamide (ZNS), primidone(PRM), clobazam (CLB),valproic acid (VPA), ethosuximide(ESM), lamotrigine (LTG), oxcarbazepine (OXC), felbamate(FBM), eslicarbazepine acetate(ESL), gabapentin (GBP), topiramate (TPM), levetiracetam (LEV), rufinamide (RFN), vigabatrin (VGB), perampanel (PER), lacosamide (LCM), pregabaline(PGB)

**SUPPLEMENTAL TABLE 5: Risk factors for antiepileptic hypersensitivity reactions in children**

| **Young age** |
| --- |
| **Aromatic antiepileptic use** |
| **Previous history of antiepileptic hypersensitivity** |
| **Family history of antiepileptic hypersensitivity** |
| **A high starting dose and rapid dose escalation,** |
| **Sodium valproate and lamotrigine combination** |
| **Multi-drug regimens including aromatics** |
| **Concurrent administration of other drugs known to affect cytochrome system** |
| **Concurrent viral infections(HIV, HHV,etc)** |
| **Carbamazapine use in the presence of HLA-B*1502 and HLA-A*3101 genotypes for certain populations** |
| **Phenytoin use in the presence of Cytochrome P450 genetic polymorphisms** |


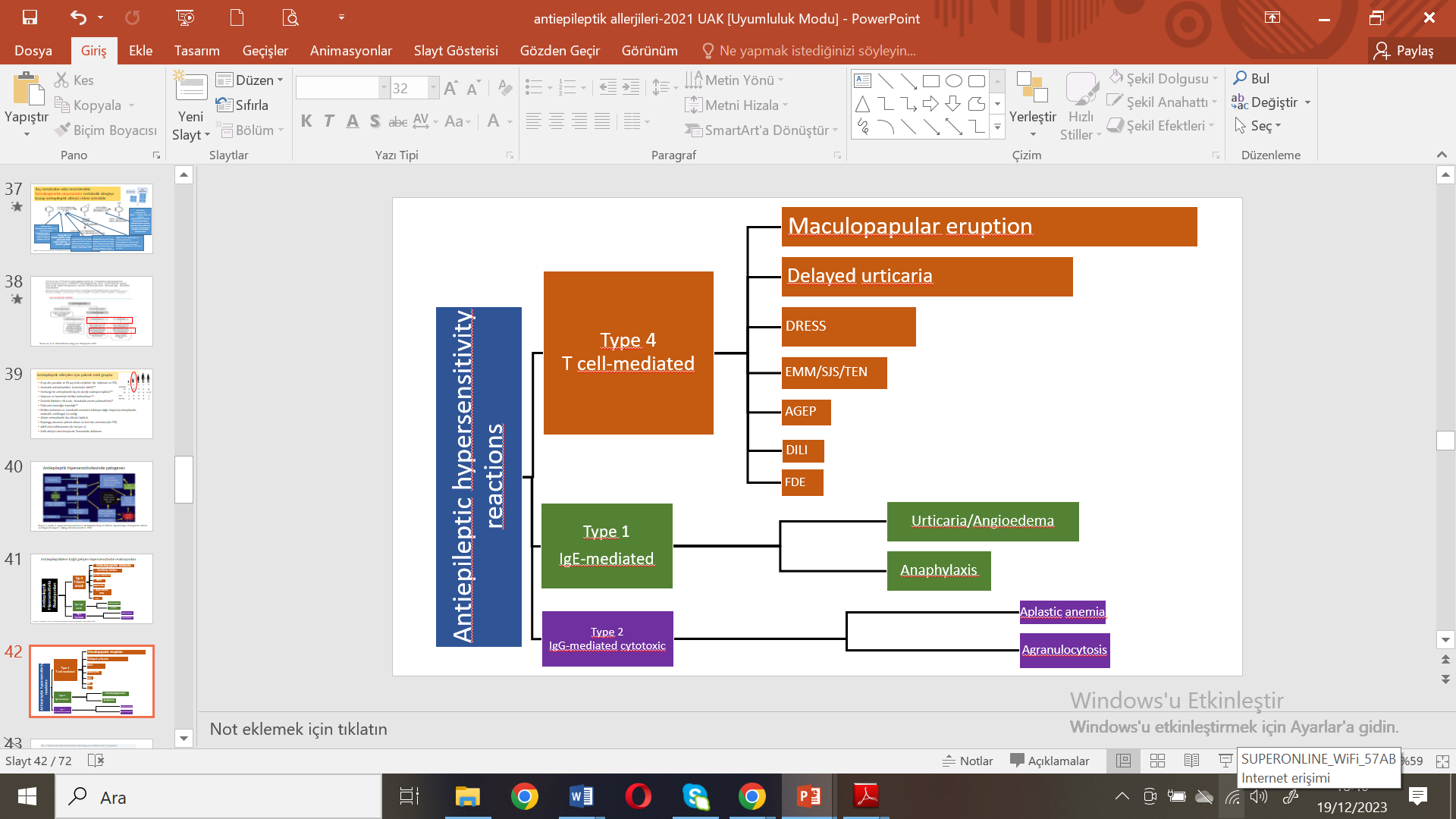


**SUPPLEMENTAL FIGURE 5** Endophenotypic classification of antiepileptic hypersensitivity reactions. Drug reaction with eosinophilia and systemic symptoms(DRESS), Erythema multiforme minor/major(EMM), Stevens-Johnson syndrome (SJS), Toxic epidermal necrolysis (TEN),Acute generalized exanthematous pustulosis (AGEP), Drug-induced liver injury(DILI), Fixed drug eruption(FDE)


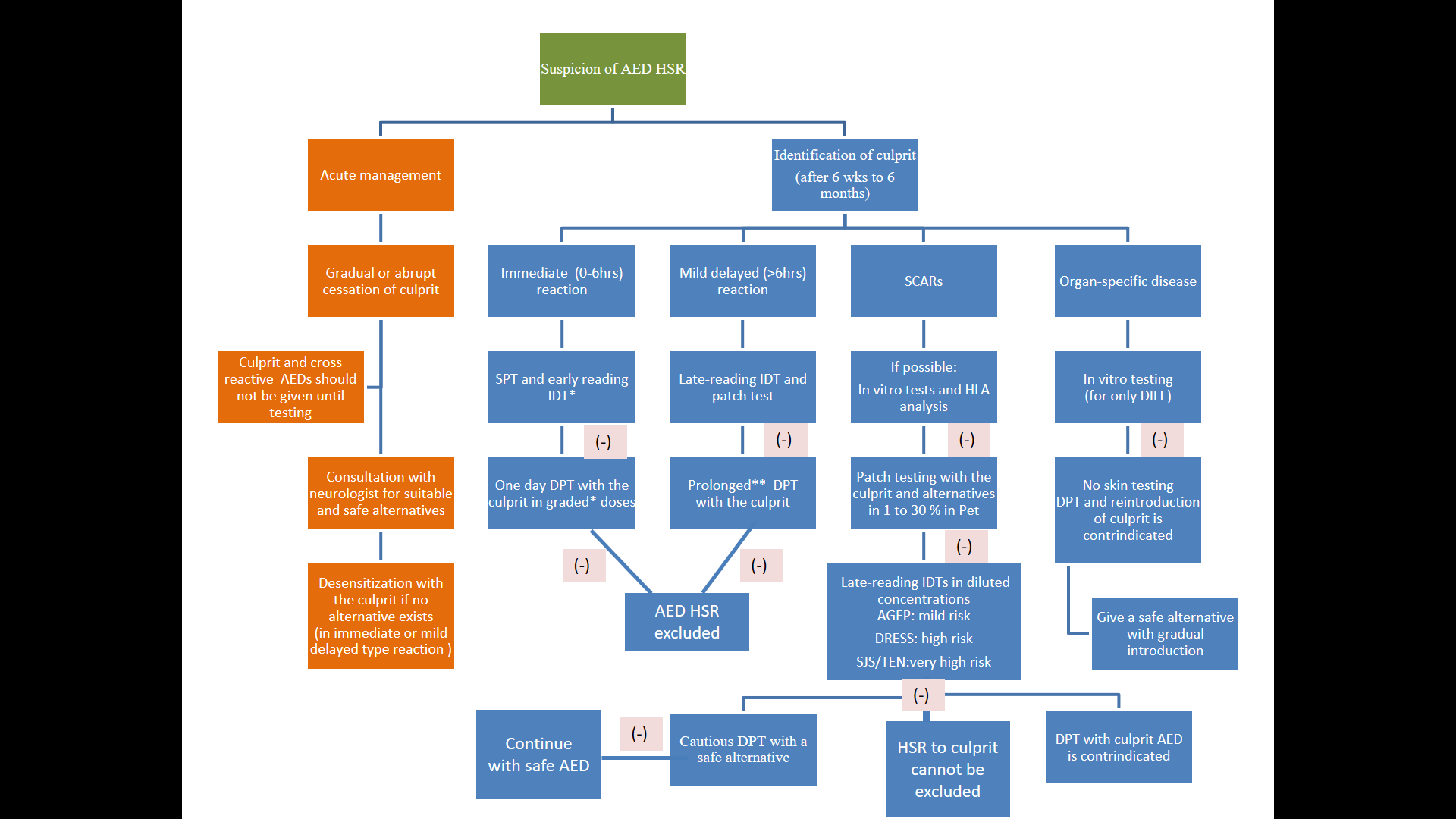


**SUPPLEMENTAL FIGURE 6:** Management of hypersensitivity reactions to antiepileptic drugs in children. A positive reaction in any step is accepted as confirmation of the HSR to culprit. Skin prick tests can be performed with all AEDs in tablet,capsule or solution forms. IDTs can be done only with drugs available in sterile parenteral preparations of LEV, PB, PHT,VPA, lacosamide and clonazepam., *Standardized maximum non-irritant prick and IDT test concentrations of AEDs are not known.**Increasing doses with maximum 5 steps. *** The duration of the DPT should be chosen according to latency period of the reaction. .(ref: 154,152,204,210-212). AED: antiepileptic drug; SPT: skin prick test; IDT: intradermal test;DPT: drug provocation test; HSR: hypersensitivity reaction; SCARs: severe cutaneous adverse drug reactions(DRESS, SJS/TEN, AGEP, E.Multiforme); DILI: drug-induced liver injury.

**SUPPLEMENTAL TABLE 6:** Studies with antiepileptic drug skin tests in children

| **Age group** | **N of cases** | **Drug tested** | **Clinical phenotype** | **Test type and conc(%)** | **Positivity rate** | **Reference** |
| --- | --- | --- | --- | --- | --- | --- |
| Mixed | 400  Meta-analysis (1950-2008) | All aromatics and VPA | All types of HSR | Patch 0.1 to 50 % pet | 20-85 %  CBZ highest  PB lowest | (Elzagallaai AA, Drug Saf. 2009) ^207^ |
| Child | 10 | CBZ, PHT, PB, LTG, RUF | DRESS  SJS/TEN | Patch 20% and 50% in pet  IDT NP  DPT NP | 2/10 | (Liccioli G, Clin Exp Allergy 2020) ^81^ |
| Child | 6 | CBZ,OXC,  LTG,PB  ESM | DRESS MPE  SJS/TEN | Patch 10% in pet | 2/6  1 CBZ-DRESS  1 ESM-SJS/TEN | (Costa Carvolho J, Contact Dermatitis 2022) ^208^ |
| Child | 44 (40 % CBZ) | CBZ,VPA,  OXC,LTG,  PB,LEV,  TPA,CLB | MPE  SCARs | Patch  5% and 10% in pet | Total: 40.9%  MPE 12/27  SCAR 6/16  CMZ showing the highest positivity rate with 62% | (Büyük Yaytokgil S, Allergy Asthma Proc 2021) ^205^ |
| Child | 100 | CBZ,VPA,  LTG,PB,  LEV,TPA,  CLB | MPE  SCARs | Patch 10 % pet | Total: 89.3%  LTG 52.1 %  CBZ 65.7%  PHB 85.7%  VPA 68.7% | (Atanaskovic- Markovic M, Pediatr Allergy Immunol. 2019) ^153^ |
| Child | 65 | CBZ,VPA,  LTG,PB | MPE  Delayed urticaria  SCARs (?) | IDT(1/100 dilution) late reading | 7/65  (10.6%) | (Atanaskovic- Markovic M, Pediatr Allergy Immunol. 2019) ^153^ |
| Child | 31 | CBZ,VPA,  OXC,LTG,  PB,LEV,  TPM,CLB | MPE  SCAR | 10% in pet | Total 58.6%  MPE 44%  SCAR 42.9%  CBZ 73.3%) VPA 27.3%  PHB 28.6%  LTG 100% | (Guvenir H, J Allergy Clin Immunol Pract. 2018) ^53^ |

Lamotrigine (LTG), carbamazepine (CBZ), phenobarbital (PB), phenytoin (PHT), oxcarbazepine (OXC), felbamate, zonisamide, primidone] or non-aromatic[sodium valproate (VPA), topiramate, levetiracetam (LEV), rufinamide(RUF), topiramate (TPM), Clobazam(CLB), Ethosuximide(ESM)

NP not performed, NA: not applicable.

**SUPPELEMTANL TABLE 7: Evaluation and management of radiocontrast media (RCM) hypersensitivity reac-tions (HSRs) in pediatric populations**

**Table S7:** Details to be assessed in the clinical history of RCM hypersensitivity reactions

| Demographic data of the patient |
| --- |
| Past medical history, reason for radiological examination, concomitant infection |
| Possible risk factors (previous cardiovascular disease, renal disease, cancer, mastocytosis, respiratory allergy, atopy, drug allergy or other adverse drug reactions, HLA-DRB1*:02, previous contrast media reaction, treatment with ACE inhibitors or beta-blockers or proton pump inhibitors, etc) |
| Concomitant drugs and pre medication if used |
| Name and brand of the ICM administered and dose |
| Administration route |
| Time interval between administration and the onset of symptoms |
| Descriptions of the symptoms |
| Analytical exams at the time of the reaction |
| Treatment required |
| Time evolution until resolution |
| Severity of the reactions and eventual sequels |
| Previous administrations and subsequent tolerance to RCM |

**SUPPLEMENTAL FIGURE 7** – Algorithm for investigation and management of patients with suspected RCM HS


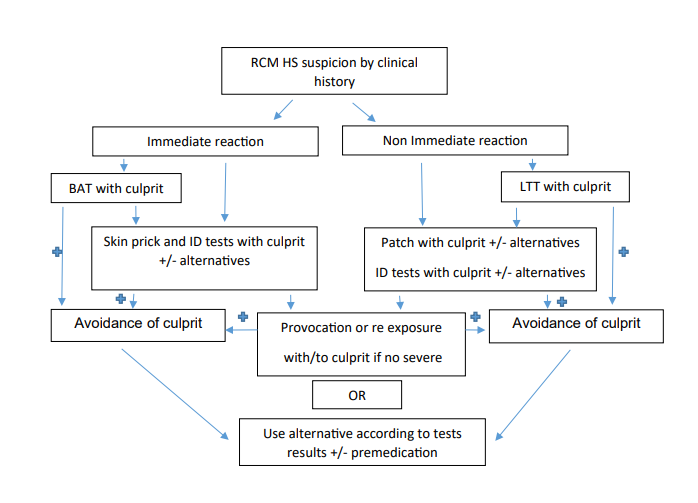


**SUPPLEMENTAL TABLE 8. Clinical features of DRESS in children. Ref(168,169,170,268,269,271)**

| Characteristics | Frequency |
| --- | --- |
| Onset of symptoms after beginning drug use | Average 15-24 days, range 1 to 112 days  antibiotics mean 13 days and with antiepileptics mean 21 days |
| Fever (> 38°C) | 90%-100% |
| Skin  Rash  Morbilliform or maculopapular rash  Generalized rash  Exfoliative rash  Erythrodermic rash  Pruritus  Edema at face or extremities or generalized | 98%-100%  70%-100%  23%-70%  27%  3%-14%  32%-84%  51%-76%, 13%-22%, 6%-9% |
| Leukocytes abnormalities  Eosinophilia  Atypical lymphocytosis  Leucocytosis | 60%-100%  50%-60%  25% |
| Thrombocytopenia | 6% |
| Anaemia | 6% |
| Lymphadenopathy | 69%-77% |
| Internal organs  Hepatitis  Splenomegaly  Kidney (interstitial nephritis, from high serum creatinine to rare renal failure)  Gastrointestinal tract (gastroenteritis, gastrointestinal bleeding, colitis)  Lung (pneumonia, acute respiratory distress syndrome)  Pancreatitis, myocarditis, pericarditis, myositis, meningoencephalitis, polyneuritis, uveitis | 80%-87%  21%  12%-26%  1%-10%  7-22%  Rare |
| Mucosal lesions (cheilitis, stomatitis, pharyngitis, conjunctivitis) | 12%-48% |
| Others: elevated CRP (56%); Skin lesions: pustules, vesicles, eczematous, urticarial, lichenoid and target-like lesions, purpura; hypogammaglobulinemia; hemophagocytic lymphohistiocytosis syndrome (24%).  Laboratory investigations: complete blood count, peripheral smear, platelets, C-reactive protein, renal and liver function tests, electrocardiogram, serology and polymerase chain reaction studies for viruses, especially human herpesvirus-6, -7, cytomegalovirus, Epstein–Barr virus, thyroid function testing, autoantibodies at baseline and during the follow-up, tests for damage of an involved organ. Skin histopathology in skin biopsy is not pathognomonic, but it may exclude other diagnosis. | |
| Mortality (hepatic failure, myocarditis, acute respiratory distress syndrome, septic and hypovolemic shock, multivisceral organ failure)  Relapses in 1‐24 months  Sequelae in 1‐24 months  Autoimmune diseases  Hypothyroidism  Diabetes mellitus  Vitiligo, systemic lupus, hyperthyroidism  Liver failure | 2.9%-5%  4.8%  8%-10.8%  3.8%  2.3%  Rare  3.1% |

**SUPPLEMENTAL TABLE 9: Diagnostic criteria for drug-induced hypersensitivity syndrome (DIHS) and DRESS that are considered different forms of the same illness.**

| Japanese consensus group for diagnosis of drug-induced hypersensitivity syndrome (DIHS) (266) | Inclusion criteria for potential case of anticonvulsant hypersensitivity syndrome (HSS)/DRESS in European Registry of Severe Cutaneous Adverse Reactions to Drugs and Collection of Biological Samples (RegiSCAR) (249) |
| --- | --- |
| Mandatory criteria | |
|  | - Hospitalization - Reaction suspected to be drug related |
| Criteria necessary for the diagnosis | |
| 7 criteria (typical DIHS) or of the five (81, 168-170, 266) (atypical DIHS). | *Three or more criteria are required. |
| 1. Maculopapular rash developing >3 weeks with a limited number of drugs | - Acute skin rash* |
| 2. Prolonged clinical symptoms after discontinuation of the causative drug |  |
| 3. Leukocyte abnormalities (>1)   - Leukocytosis (>11 x10^9^/L) - Atypical lymphocytes (>5%) - Eosinophilia (>1.5 x10^9^/L) | Blood count abnormalities   - Lymphocytes above or below the laboratory limits* - Eosinophils above the laboratory limits (in percentage or absolute count)* - Platelets below the laboratory limits* |
| 4. Fever >38°C | - Fever above 38 C* |
| 5. Liver abnormalities (ALT >100 U/L) or other organ involvement, such as renal involvement | - Involvement of at least one internal organ* |
| 6. Lymphoadenopathy | - Enlarged lymph nodes at at least two sites* |
| 7. Human herpes virus 6 reactivation |  |

**SUPPLEMENTAL TABLE 10. Scoring system of the European Registry of Severe Cutaneous Adverse Reactions to Drugs and Collection of Biological Samples (RegiSCAR) for diagnosing HSS/DRESS cases as definite, probable, possible or no case** (267). Total score < 2, no case; 2–3, possible case; 4–5, probable case; > 5 definite case. ^a^After exclusion of other explanations:1, one organ; 2, two or more organs. *HAV, hepatitis A virus; HBV, hepatitis B virus; HCV, hepatitis C virus.

| Score | -1 | 0 | +1 | +2 |
| --- | --- | --- | --- | --- |
| Fever ≥ 38.5°C (core) or ≥ 38°C (axillary)  Enlarged lymph nodes nodes (≥2 Sites, >1 cm)  Eosinophilia  Eosinophils  Eosinophils,if leucocytes < 4.0 x10^9^ L^-1^  Atypical lymphocytes  Skin involvement  Skin rash extent (% body surface area)  Skin rash suggesting DRESS  Biopsy suggesting DRESS  Organ involvement^a^  Liver  Kidney  Lung  Muscle/heart  Pancreas  Other organ  Resolution >15 days  Evaluation of other potential causes  (Antinuclear antibody,blood culture, Serology for HAV/HBV/HCV*, Chlamydia/mycoplasma) if none positive and and > 3 of above negative | No/Unknown  No  No  No/Unknown | Yes  No/Unknown  No/Unknown  No/Unknown  No/Unknown  Unknown  Yes/Unknown  No/Unknown  No/Unknown  No/Unknown  No/Unknown  No/Unknown  No/Unknown  Yes | Yes  0.7–1.499x10^9^ L^-1^  10–19.9%  Yes  >50%  Yes  Yes  Yes  Yes  Yes  Yes  Yes  Yes | >1.5x10^9^ L^-1^  >20% |

**SUPPLEMENTAL TABLE 10:** Unique Challenges and Practical Considerations in Pediatric Patients with Drug Hypersensitivity Reactions to Chemotherapy and Biologics

| **Aspect** | **Impact on Management** | **Proposed Solution** |
| --- | --- | --- |
| Clinical History | •Limited patient recall.  •Parental discordance.  •Parental exaggeration.  •Children may struggle to express themselves. | •Standardize recording procedures in infusion centers and obtain tryptase and IL6.  •Involve caregivers; employ visual aids for clarity. |
| Skin Testing | •Logistical challenges and pain.  •Poor standardization in children. | •Collaborate with pharmacy and occupational health.  •Select tests carefully.  •Adapt testing methods for pediatric tolerance.  •Follow guidelines; use non-irritating concentrations.  •Interpret cautiously. |
| In vitro Testing in Children | •Slow turnaround times.  •Lack of validation. | •Approach in vitro results with caution.  •Integrate clinical context and additional diagnostic methods. |
| Drug Challenge | •High-risk procedure.  •Potential trauma of a reactive challenge vs drawbacks of unnecessary RDDs.  •Family expectations and nocebo effects.  •Observer bias in test assessment. | •Pediatric intensive care unit.  •Limit challenges to patients with favorable risk assessment.  •Prioritize patient safety.  •Non-essential challenges can be delayed and offered after empirical RDD.  •Manage patient expectations, standardize interpretation, and mitigate false positives (blind, placebo-controlled, team discussion, expert input, and considering revisiting inconclusive results later on). |
| Rapid Drug Desensitization | •High-risk procedure  •Children may struggle to stay still during lengthy procedures.  •Volumes might be different. | •Dedicated spaces or pediatric intensive care unit.  •Checklists.  •Inform caregivers; tailor settings for pediatric needs.  •Personalize RDD protocol bag volumes.  •Emergency treatment calculated for child’s age and weight. |
| Setting up the Service | Address complexities; involve multidisciplinary teams. | •Plan appropriately and involve the right stakeholders.  •Engage diverse expertise and patients.  •Allergists, pediatricians, nurses with pediatric skills, pharmacists, lab/immunology, intensive care, and management.  •Engange adult allergy team.  •Follow international guidance.  •Redesign care.  •SOPs.  •Optimize and standardize procedures; internal validation of process and techniques.  •Identify and measure quality indicatiors.  •Engage in training and quality improvement. |

Legend: DHR, drug hypersensitivity reaction; RDD, rapid drug desensitization; SOP, standard operating procedure

**SUPPLEMENTAL TABLE 11:** Unresolved Challenges and Opportunities for Optimizing Pediatric Allergy Management in Reactions to Chemotherapy and Biologics

| **Unmet Need** | **Impact on Care** | **WAO’s Research Call to Action** |
| --- | --- | --- |
| Diagnostic techniques | •Potential variability and uncertainty in clinical practice.  •Misdiagnosis  •Unnecessary allergy labels  •Unsafe re-administration | •Epidemiological studies, including endophenotyping  •Liaising with infusion centres to obtain biomarkers during initial reactions  •Studies to determine non-irritating concentrations and optimal techniques for skin testing in children  •Validation of in vitro tests for pediatric use  •Validation of drug challenge strategies |
| Scarce Published Studies on RDD in Children | •Limited evidence for pediatric RDD protocols.  •Potential variability and uncertainty in clinical practice. | •Multicentric studies exploring RDD in children.  •validating pathways  •validating protocols  •demonstrating effects on disease outcomes  •in vitro data  •quality of life  •cost-effectiveness  •identifying quality indicators  •Involve patients and patient advocacy groups in study design and dissemination of findings. |
| Lack of comprehensive implementation and management guidelines | •Difficulty in managing breakthrough symptoms in children during RDD.  •Potential compromise of treatment effectiveness and safety. | •Publication of nursing-specific detailed SOPs for RDD.  •Publication of pharmacy-specific SOPs for RDD  •Guidance on safe handling of chemotherapy in allergy departments.  •Comprehensive guidelines for managing breakthrough symptoms during RDD in children.  •Education materials for patients and caregivers on recognizing and reporting symptoms promptly.  •National-level efforts to establish standardized evidence-based guidelines for children undergoing RDD. |
| Psychological and Social Impact of Drug Allergy | •Negative psychological and social impacts on pediatric patients and families due to drug allergy.  •Potential impact on treatment adherence and outcomes. | •Evaluate the psychological and social impacts of drug allergy and RDD in children.  •Implement support programs and resources for patients and families, including counseling and peer support groups.  •Involve patient associations in raising awareness and advocating for resources. |
| Debated Use of Premedication in Children | •Lack of consensus on premedication strategies for children undergoing DDS.  •Variability and uncertainty in clinical practice. | •Clinical trials to identify the best strategies  •Systematic research in personalization and precision medicine  •Involve patient associations in developing guidelines and providing input on patient preferences and experiences. |
| Lack of Widespread Implementation | •Patient Inequality  •Allergic patients receive different care depending on access to allergy services  •Unsafe management by non-experts  •Unnecessary avoidance  •Unsafe rechallenge | •Allergy societies and groups to liaise with oncology and other relevant stakeholders at a national level.  •Involve patient associations in decision-making processes.  •Implement allergy-led training programs for healthcare providers.  •Specify training needs in curriculums for allergists, allergy nurses, allergy pharmacists, and allergy champions.  •Creation of allergy hubs that can be accessed at least regionally  •National-level accreditation programs and minimum quality standards |

Legend: RDD, rapid drug desensitization; SOP, standard operating procedure

**SUPPLEMENTAL TABLE 12: Data regarding suspected/proven causes of peri-operative hypersensitivity (POH) from paediatric studies. Grades refers to Ring and Messmer classification**. ^(343)^

| Geographical area | Dates | No. and type of cases | Causes |
| --- | --- | --- | --- |
| UK, France, US ^(356)^ | 2006-2016 | 6 centres, 29 cases grade 3 and 4 | Culprit identified by positive testing in 15. NMBA (8), Antibiotics (3), omnipaque (1), latex (1), laxative (1), Midazolam (1). |
| Australia ^(357)^ | 2015-2019 | 1 centre, 15 cases grade 2 and above | Culprit identified in 5/8 of the 15 who completed testing. NMBA (2), Antibiotics (3). |
| Turkey^(355)^ | 2007-2019 | 1 centre, 50 cases, grades 1-4. | Culprit identified in 14/50 patients, 3 of these also co-sensitised to latex. NMBA (8), midazolam (3), ketamine (2), propofol (1). |
| Turkey^(362)^ | 2019-2021 | 1 centre, grades 1-4 | 29 cases. 1 definite anaphylaxis (defined as positive skin test and tryptase), 5 possible. Overall, 9 patients had positive testing. Co-sensitisation present in 5. In only 1 patient did the authors feel temporal relationship was clear (cefazolin was the culprit). Positive tests NMBA (3), sugammadex (1), latex (5) (co-sensitised in all 5 and 4/5 undertook and tolerated challenge) ketamine (1), midazolam (1) chlorhexidine (4(but IgE negative (co-sensitised in 3)) cefazolin (1), ketamine (1). |
| France ^(351)^ | 1989-2001 | 1 centre 68 children, grades 1-3 | Culprit identified in 51/68 children with co-sensitisation present in 11 cases, NMBA (31), latex (14), colloids (7), opioids(5) (Alfentanil (2), sufentanil (2), fentanyl (1)), hypnotics (6)(propofol (2), thiopentone (1), ketamine (1), midazolam (2)). |
| France and Belgium ^(361)^ | 1991-1992 | 38 centres, 21 children, grades 2-4 equivalent | 21 cases, (IgE mediated reactions only included) 20/21 cuprits identified: latex (16), NMBA (2), penicillin(1) and contrast (1). |
| Europe^(360)^ | 2014-2015 (2 week period each centre) | Multi-centre Europe (261 centres, 33 countries). Not solely POH-focused. Assessed critical events in paediatric anaesthesia. | 3 cases of anaphylaxis, 2 of which were confirmed: erythromycin(1) and latex (1) and one suspected, neostigmine (1) |
| US^(352)^ | 2010-2017 | 29 institutions across US documented anaphylaxis within database of anaesthetic cases. | 62 cases of anaphylaxis, proposed suspected triggers (21 were known to have had allergy testing) were latex (6), contrast media (8), antibiotics (14), NMBA (11), opioid analgesics (8), protamine (4), IV anaesthetic agent (3), thrombin gel (3), aminocaproic acid (3), NMBA reversal agent (1), 5% albumin (1), hypnotics (1), tranexamic acid (1), blue dye (1), platelets (2), whilst 2 had an unknown trigger, and 7 had more than one suspected trigger. |

**SUPPLEMENTAL FIGURE 10:** Due to the wider range of procedures for which anaesthetics are undertaken in children compared to adults, information may need to be collated from different specialties. All referrals should include an anaesthetic chart and referral form (NAP6 ^(358)^). Within the allergy team, it is important to provide a multidisciplinary approach to facilitate testing e.g. with support from play specialists or psychologists, and more than one appointment may be needed to complete the tests.


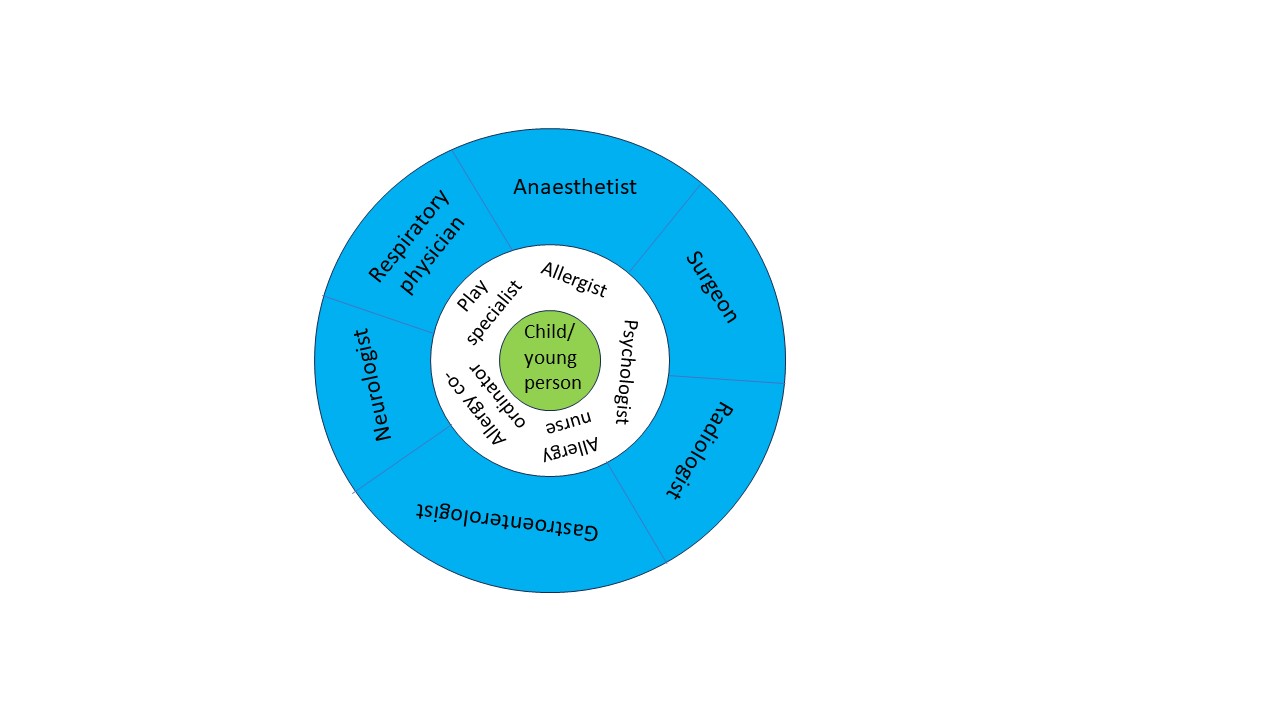


**SUPPLEMENTAL TABLE 13:** Checklist to ensure adequate information transfer, and support for the family after POH testing. ^(358)^

| **Checklist at completion of POH testing** | **Completed (y/n)** |
| --- | --- |
| Updated allergy information on hospital electronic charts |  |
| Letter to GP & all relevant healthcare practitioners detailing allergy tests undertaken and ongoing advice |  |
| Advice given to child/young person and family to carry a medical ID jewellery/card |  |
| Ideally a digital copy of the report given to family with advice to share this with the child/young person as they become independent. |  |
| Address psychological morbidity from POH and refer as needed for psychology support |  |
