# Supplementary material for: Drug hypersensitivity reactions in children in clinical practice: A WAO Statement
Source: World Allergy Organ J. 2025 Aug 29;18(9):101087. doi: 10.1016/j.waojou.2025.101087 (PMC12419032; doi:10.1016/j.waojou.2025.101087)
Supplement: Multimedia component 2 [file mmc2.docx]

# SUPPLEMENTAL APPENDIX 1

Pediatric Allergy Technical Area for Diagnostic and Therapeutic Procedures: "*Breaking Views*"

Authors: Alvarez-Cuesta E, Berges-Gimeno P, Guzman-Melendez MA, Madrigal-Burgaleta R

**Introduction: Designing an Outstanding Pediatric Allergy Diagnostic and Therapeutic Area**

The practice of Pediatric Allergy and Clinical Immunology relies on high standards of clinical excellence (1-5). A critical component of this practice is having an exceptional diagnostic and therapeutic area—often considered the cornerstone of an Allergy and Clinical Immunology Division. To draw an analogy, the technical area for an allergist is as crucial as an operating room is for a surgeon.

When developing, redesigning, or updating a Pediatric Diagnostic and Therapeutic Area, several key questions should be addressed:

1. Does our current pediatric technical area meet the quality and safety standards of the 21st century?
2. Are the procedures we have sufficient to answer the questions posed by our patients?
3. Does the area provide optimal conditions, including comfort and safety for children, adolescents, and their families, through its design, equipment, and overall experience? Are we proud of it?
4. When was the last time we reassessed, redesigned, or refined our procedural area?
5. What adjustments should we make to improve its safety?
6. When did we last challenge and break away from established paradigms?

Defining the essential elements for redesigning, creating, or updating a pediatric allergy diagnostic and therapeutic area is crucial. Below is a summary of key considerations, which we refer to as the "10 P's":

1. **Purpose & Priorities**
2. **People**
3. **Parent**
4. **Powerhouse of the Division of Allergy**
5. **Process**
6. **Performance**
7. **Price**
8. **Perspective**
9. **Projects**
10. **PubMed**

# Purpose & Priorities - Core Objectives and Strategic Priorities for the Allergy Division

The staff of the Allergy Division and the technical area must have clarity about their responsibilities. They will work with well-defined objectives and priorities aligned with the institution's mission, vision, values, and the cultural context of both the country and the organization.

Planning is essential for establishing a long-term vision and a clear roadmap. This will facilitate decision-making and efficient resource allocation, while allowing flexibility in response to environmental changes. Without planning, we risk being caught in a maze of uncertainties and doubts about the best course of action.

The seven primary objectives should be:

1. **Patient-Centered Care:** The foremost objective is to place pediatric and adolescent patients at the center of our focus. We must dedicate ample time to addressing the concerns and problems raised by both the patient and their referring physician. **The patient should always be our priority and our primary focus** (6-27).
2. **Patient Satisfaction:** We are committed to meeting patient needs by offering personalized strategies that cater to their unique requirements and goals. Medical services must be of the highest quality, safety, and empathy, with clear communication about available options for the patient.
3. **Excellent Technical Area:** It is our responsibility to ensure that allergists work in a technically advanced diagnostic and therapeutic area, with a comprehensive service portfolio to benefit our patients.
4. **Prevention:** We will adopt a proactive approach to patient care. This means anticipating potential issues during diagnosis or treatment, minimizing risk factors, and mitigating consequences.
5. **Efficiency:** Ensuring operational effectiveness is crucial for maintaining current success ("it guarantees the present").
6. **Innovation:** We will drive future growth and quality through continuous innovation, which is beneficial for patients, but also attract and retains high-profile staff ("it guarantees the future").
7. **Teaching:** We view teaching as a duty, a commitment, and an expression of generosity.

# People:

- 1. Leadership Roles and Responsibilities

The Allergy Division's success hinges on effective co-direction, exemplifying "Team Leadership." Key roles include:

- **Division Lead:** Focuses on overall management of the Allergy Division.
- **Technical Area Lead:** Concentrates on the technical aspects within the Allergy Division.

It is crucial to balance allergists with technical expertise and those with administrative skills. Co-leaders must ensure seamless integration of the allergy specialty's operations for the benefit of patients and their families. They should be committed, open to peer advice, and proactive in adding value to the organization. Effective delegation and horizontal collaboration are essential.

- 1. Qualifications and Skills for the Technical Area Lead

The Technical Area Lead should be a "full allergist" of recognized prestige with:

- Advanced technical knowledge and experience in diagnostic and therapeutic allergy techniques.
- Proven leadership skills and commitment.
- This allergist will be a part of the allergy division but dedicated exclusively to the technical area.
- They will be responsible for ensuring that an allergist with technical solvency is always present in this area during all twelve hours of daily activity.
- A CV reflecting successful project involvement, academic experience abroad, and specialized training.

Technical expertise alone is insufficient; a strong attitude towards problem-solving, adaptation, and continuous improvement is vital. Qualities such as critical thinking, culture-creating abilities, empathy, listening, not accepting dogmas, and flexibility are essential.

Consider appointing a physician with dual certification: a "full allergist" and a "pediatric intensivist" or equivalent.

Consider that clinical-focused allergists will provide general and specialized consultations aligned with their projects. One may want to seek out candidates that can show clarity in project development and emit 'the energy of the project director'.

- 1. Team Collaboration and Multidisciplinary Teams Approach

Working as a team, creating and belonging to multidisciplinary teams (learning, exchanging resources) must be the "modus operandi". The importance of this work-place ethos is cleverly explained by Simon Sinek: "**The ability for a group of people to do remarkable things hinges on how well those people can pull together as a team**."

Adopting a team-based approach is fundamental. Multidisciplinary teams, led by the Technical Area Lead, should include nurses, allied health professionals, and other personnel. This collaborative model supports complex case discussions and decisions regarding technical equipment, clothing needs, and pharmacological resources.

Local staffing provisions may greatly differ from country to country. However, an additional co-leading key role should work in sync with the core leadership whenever possible:

- **Lead Nurse:** Is responsible for overseeing nursing staff, ensuring high standards of patient care, managing training and quality control, and coordinating with other healthcare professionals to maintain efficient and effective operations within the technical area.

Nurses play the key role in the running of the technical area. The lead nurse should agree with the technical area lead on special training, periodic training sessions, continuous updating, simulation training, quality improvement projects, and other requirements.

The nursing team in the technical area should have time and space to perform other nursing roles, such as patient education tasks or nurse-led clinics.

- 1. Commitment to Professional Development

The Allergy Division's co-leaders must be actively involved in professional development, ensuring access to ongoing training and cutting-edge resources.

- 1. Passion and Commitment

We seek allergists who are passionate about the specialty, driven by dedication rather than merely accumulating certificates. True commitment involves a deep connection to patients, teamwork, and the institution, which fuels effort, sacrifice, and achievement of ambitious goals.

# Parent – Understanding the Foundational Aspects: Core Framework for Integrated Management and Team Dynamics in the Allergy Division

**Let's define and analyze the Allergy Division, focusing on team collaboration and participation in multidisciplinary teams.**

When structuring an allergy division, it is essential to consider the broader framework within which the specialty will develop, adapting accordingly. Key questions include: Will the Allergy Division be based in a university hospital (tertiary referral center) or in a secondary or community hospital? What services are planned for this allergy division? What are the prevailing cultures within the hospital, the country, the affiliated university, and even the local population and city where the Allergy Division will be situated? What is the prevalence of allergic conditions in this region? Additionally, what is the hospital’s budget, and what portion is allocated specifically for the Allergy Division? These considerations are fundamental to effective planning and operational alignment.

The Allergy Division will provide (1-2):

- General outpatient clinics
- Specialized monographic clinics, essential for translational research projects
- Dedicated beds within a designated hospital area
- Access to intensive care unit beds when needed
- A comprehensive diagnostic and therapeutic technical area

This outstanding technical area, a centerpiece of the Allergy Division, should be ideally located adjacent to the allergy clinics area and managed by a highly regarded allergist with advanced technical expertise, as previously outlined.

It is crucial to operate cohesively and harmoniously. We might ask: If our Allergy Division were an orchestra, would it be in tune? Do all members follow the same score, or are they working in isolation? Are innovative ideas obstructed, or are they supported and developed in tune with the rest of the allegy division?

Additionally, the Allergy Division and its technical area will interact with other hospital services, particularly:

- The Pharmacy Department for preparing solutions for skin tests, patch tests, drug challenges (or provocation tests), and desensitizations
- Clinical staff from related specialties and referring specialties

It is essential for the entire hospital, especially the mentioned specialties, to stay updated on continuous improvements and new procedures in the technical area. This collaboration will ultimately benefit patients, promote the specialty within the hospital, and extend its impact to other institutions, as innovation always transcends boundaries.

# Powerhouse of the Division of Allergy:

***Our goal is to establish an outstanding Diagnostic and Therapeutic Technical Area.*** ***This area will also integrate a Clinical Research Unit. Additionally, we aim to develop an excellent portfolio of services.***

See **FIGURE 1** for a graphic example of an Allergy Technical Area.

See **TABLE 1** for a risk assessment matrix for an Allergy Technical Area.

See **TABLE 2** for information on quality indicators for an Allergy Technical Area.

- 1. Design and Integration of the Allergy Technical Area

The diagnostic and therapeutic technical area of the allergy division should serve as a central hub for managing rapidly increasing, often severe, and complex pathologies. It should encompass the following functions (1, 2, 5):

- Patient care (diagnosis, delabeling, and therapy)
- Education
- Research
- Prevention
- Dissemination of knowledge

The area should epitomize continuous improvement in quality, safety, and efficiency. Integrating the clinical research unit within this technical area will enhance both allergy research and clinical care. If direct integration is not feasible, placing the unit in close proximity is preferable.

- 1. Patient Care and Facilities

The technical area should prioritize patient-centered care, focusing on the satisfaction and well-being of children, adolescents, and their families according to local customs. Patient safety, healthcare education, and comprehensive care must be paramount, ensuring access to high-quality healthcare with warmth and compassion. Essentially, patients should receive the best possible and personalized care, both diagnostically and therapeutically. The primary focus should be on patients with severe conditions, high complexity, and high risk.

To facilitate patient access to our services, the allergy division should ideally operate 12-14 hours a day, six days a week. It should be located adjacent to the allergy outpatient consultation area and as close as possible to the Pediatric Intensive Care Unit.

Additional facilities should include:

- A teaching room
- Nursing consultation rooms
- A multipurpose workspace

Optimal signage within the hospital is essential for ease of navigation.

- 1. Design Considerations for the Allergy Technical Area: Dimensions, Layout, and Aesthetic Integration

To determine the dimensions and modules of the technical area, we must consider several factors related to the increasing prevalence of allergy-related conditions and the characteristics of our patient population. These factors include:

- Prevalent diseases in the catchment area
- Referrals from other hospitals
- Patient needs
- Disease complexity
- Services provided
- Departmental forecasts
- Future programs and programs under development

All stakeholders should contribute to decision-making to ensure a comprehensive approach.

- - 1. Design Priorities and Considerations

When designing the technical area, the following priorities should be emphasized:

- **Accessibility:** Ensure easy access for both patients and staff.
- **Spaciousness:** Provide ample space to accommodate patient care and movement comfortably.
- **Functionality:** Design the area to support efficient operations and care delivery.
- **Natural Lighting:** Maximize daylight to enhance patient and staff comfort.
- **High Ceilings:** Consider high ceilings to create an open and airy environment.

Efficient spatial flow is crucial for safety. Ensure that corridors and doors are wide and unobstructed to facilitate smooth movement. Additionally, factor in local customs, traditions, and conventions to ensure the design meets the needs of the community.

- - 1. Interior Design Guidelines

To achieve functionality and balance in the technical area, consider the following design elements:

- **Symmetry:** Create a sense of order and elegance within the space that should instil calmness.
- **Proportions:** Ensure that scale, size, and shape are well-suited for the space.
- **Anthropometric Measurements:** Use measurements based on human dimensions to achieve a functional and visually appealing layout.
- **Colors and Lighting:**
  - Apply a neutral base color to approximately 60% of the space.
  - Use a darker color for 30% of the space and a soft, luminous contrast color for the remaining 10%.
  - Prioritize natural light in all rooms to enhance comfort.
- **Adding a touch of "Feng Shui":** Incorporate principles of harmony between inhabited space and the people within it, and orderliness to improve the space's ambiance.
  - 1. Space Layout Recommendations

For the technical area, a rectangular layout that is wide and long is preferred (1, 2). An island in the center of the rectangle can efficiently house:

- Patient reception
- Administrative office
- Clean and dirty areas
- Storage room
- Restrooms for patients and accompanying persons
- Staff restrooms
- Refrigerators and freezers equipped with remote temperature monitoring systems

These changes aim to improve the clarity, organization, and readability of the text, making it more accessible and easier to follow.

- 1. Room Types and Functions

The technical area will consist of various types of rooms:

- **Waiting Rooms**
- **Action Rooms**
- **Monitoring / Observation / Recovery Rooms**
- **Premium Rooms**
- **Iconic Rooms**

Monitoring/follow-up rooms will be subdivided based on security levels:

- **Green Rooms:** For minimal risk patients
- **Yellow Rooms:** For moderate risk patients
- **Red Rooms:** For high-risk patients

A fourth level, represented by the color white, may be added if needed. This classification allows for resource optimization and appropriate room allocation based on the type of procedure and patient risk.

- 1. Premium Rooms

"Premium Rooms" are multifunctional rooms designed for both action and strict, close monitoring. They are equipped for high-dependency care (an advanced level of intermediate care/monitoring, not reaching intensive care needs). These rooms represent a step-up in level of care compared to the red rooms, which will be described later.

Premium rooms are multifunctional spaces designed for both action and close monitoring. They are equipped for high-dependency care, representing a step up from the red rooms described later. Premium rooms will be assigned to patients requiring special supervision due to extremely high personal risk, complex diagnostic or treatment procedures, or severe illness. All patients in these rooms will be on beds, with the number of beds determined locally based on specific needs (1,2,6-10)

- - 1. Key Elements for Premium Rooms
- **Nursing Staff:** There should be one nurse for every one or two patients, depending on complexity and local guidelines. The nursing staff must be highly qualified in allergological procedures and managing severe, complex pediatric patients.
- **Trained Allergist:** A well-trained allergist must always be available and located in close proximity.
- **Appropriate Technology:** The facility must be equipped with:
  - Vital signs monitors (one for each patient)
  - A central vital signs monitoring station
  - A pediatric and adolescent resuscitation cart (with cannulas, intubation equipment, pediatric probes, etc.)
  - A defibrillator

These monitors will continuously and non-invasively record heart rate, respiratory rate, arterial oxygen saturation, and blood pressure.

- **Additional Needs:** This includes equipment for treating anaphylaxis, oxygen and suction systems, and multi-step pumps, amongst other local needs. Both the nursing staff and the allergist will closely monitor the patient throughout procedures. Available resources will limit the extent of care we can provide our patients.
- **Managerial considerations:**
  - The type of procedures performed in premium rooms include higher risk procedures such as drug or food challenges/provocation tests and desensitizations (11,12-15-20,22,23).
  - Additionally, this room can accommodate other procedures classified as specially high-risk, for example, bee venom immunotherapy (18) in a child with mastocytosis, or allergen-specific immunotherapy in a patient that has possibly experienced anaphylaxis.
  - All these procedures are carried out as day case activity, even if some patients may have to stay on their beds for up to 12 to 14 hours each day in this room. This area is not designed as a 24 hour ward for patients to stay overnight.
    1. Room Location and Design

Premium rooms should be located at a corner of the technical area's rectangle, spanning both sides to facilitate multifunctional use. Inside the Premium rooms, there should be:

- A restroom for patients
- A well-equipped changing area
- A work zone for nursing staff with a sink, telephone, computer, printer, medication preparation area, waste classifier, and containers for hazardous or bio-contaminated materials

Given that patients may spend several hours in these rooms, it is recommended to provide Wi-Fi and television screens for added comfort. Patients should be advised to bring quiet entertainment.

Premium rooms could also serve as the core room for the clinical research unit (for Phase I, II, III studies) when not used for standard clinical care.

- 1. Observation/Follow-Up/Monitoring Rooms

These rooms, categorized by risk level:

- Green Rooms: Minimal risk
- Yellow Rooms: Moderate risk
- Red Rooms: High risk

Each room's equipment and personnel assignment will vary based on its risk category. Ensuring safety and preventing anaphylaxis are critical at all times.

- 1. Red Rooms

Red rooms are designated for high-risk patients (2, 20, 22). Procedures and supervision will be managed by one specialized nurse for every 2 to 3 patients, with an allergist readily accessible. These rooms will be equipped with:

Comfortable, spacious recliners (one per patient)

- A vital signs monitor for every 3 patients
- A certified resuscitation stretcher
- Multiple oxygen and suction outlets
- Sphygmomanometers and pulse oximeters
- At least one highly qualified nurse present at all times
- Allergist readily available
- Appropriate anaphylaxis kit
- A nursing work area with a sink will also be included
  1. Yellow Observation Rooms

Yellow rooms will serve patients with medium risk (2, 21, 24-27). They should be located near red and premium rooms and restrooms to minimize patient movement. Furnishings will include comfortable chairs or armchairs and proximity to nurses and physicians.

In some cases, particularly in the pediatric sector, integrating red and yellow rooms may be preferable for a sense of expansiveness. This design facilitates flow between spaces without doors and enhances functionality. Local decisions will determine this design choice. To improve visual expansiveness, walls and flooring should use similar colors and materials. "Safety First" and the prevention and management of anaphylaxis are paramount. If this integration option is chosen, it would be advisable to adjust algorithms, protocols, and all other necessary factors.

**Figure 1: A schematic design inspired by Ramon y Cajal University Hospital's Allergy Division's Technical Area for Diagnostic and Therapeutic Procedures.**

A) Allergy Technical Area (TA) B) Adjacent Spaces to the South of the TA
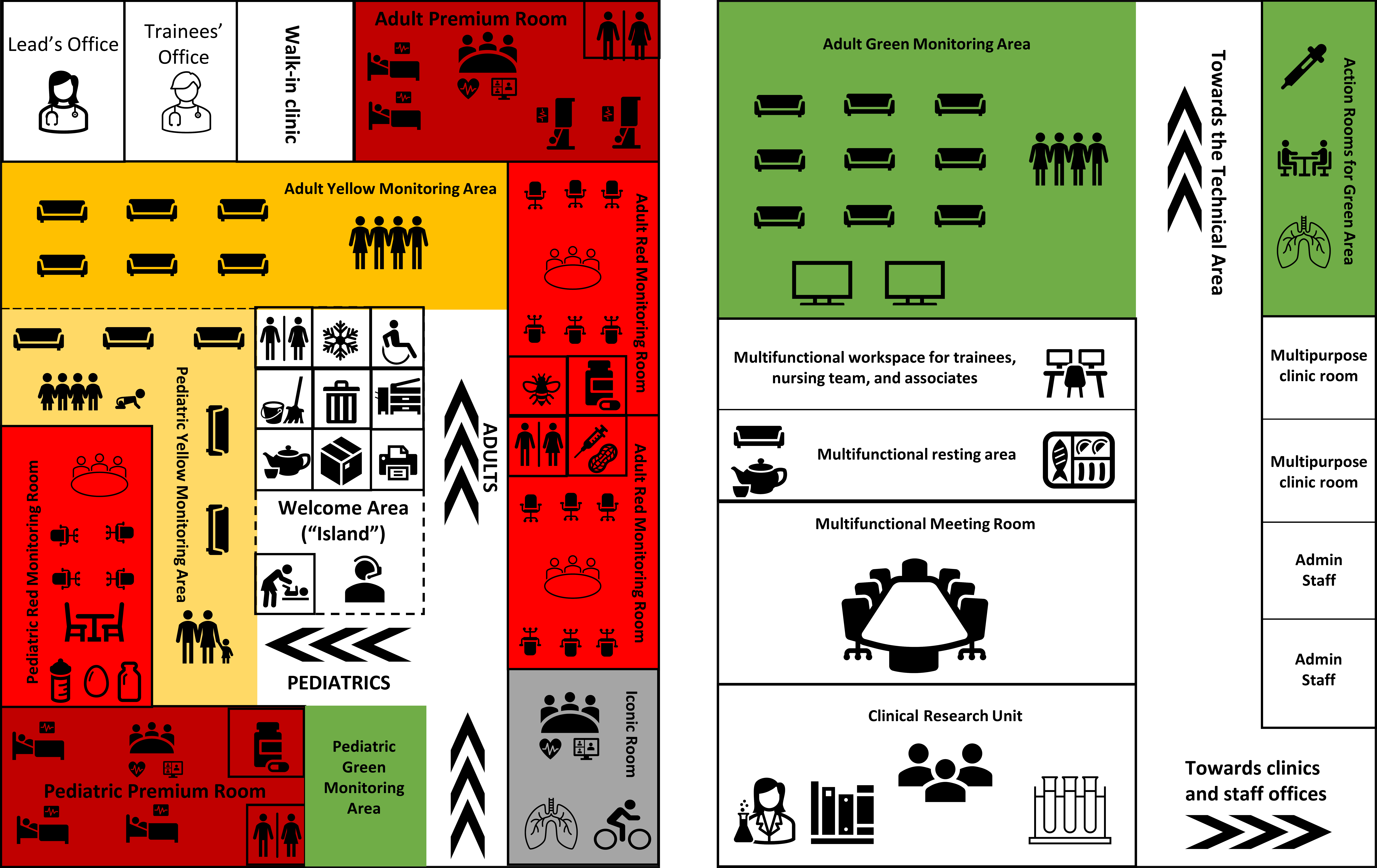


LEGEND: Part A) shows the blueprint for the Technical Area. Part B) show the immediately adjacent areas. Please, be aware that this schema is only a design inspiration for the reader. This figure is not trying to reliably reproduce the Ramon y Cajal University Hospital's allergy Technical Area blueprint. For instance, the dimensions of the rooms are not accurate and have been modified for the sake of clarity. In addition, some rooms or their location might be still a design idea not yet realized.

*Modified with permission from: Alvarez-Cuesta et al. Standards for practical intravenous rapid drug desensitization & delabeling: A WAO committee statement. World Allergy Organization Journal (2022) 15:100640. http://doi.org/10.1016/j.waojou.2022.100640*

- 1. Additional Considerations and Recommendations

It is crucial to plan the technical engineering aspects (e.g., electrics) of this high-dependency unit in collaboration with hospital technical staff. Additionally, consider:

- "Walk-in clinics" to facilitate both technical area and Clinical Research Unit operations
- Work offices for staff and trainees
- A multifunctional room for nursing staff rest
- A dining area for clinical trial volunteers

Adhere to the hospital's standards for technical area design. For specific procedures requiring instrument cleaning and sterilization, plan for a room with:

- Adequate ventilation
- A large sink with hot and cold water
- An air extraction system for disinfectants

Depending on the designated function of iconic rooms (e.g. bronchial challenges), ventilation systems providing 12 to 14 air changes per hour with roof exhaust or negative pressure may be required.

A translational research laboratory should be closely connected to the technical area.

In medium and low-complexity hospitals or clinics, designing and equipping the Allergy Division's technical area to the highest possible standard, including a clinical research area, is desirable but may not always be feasible.

# Process - Enhancing Workflow and Efficiency in Pediatric Allergy Technical Areas

Establishing clarity on required actions and implementation strategies is crucial for optimizing workflow. We propose setting clear standards and utilizing standardized procedures to minimize variability. This approach will streamline processes and foster a positive cultural shift within the organization.

Following the principle of "Think globally, act locally" (28), our focus will be on ensuring that the Allergy Division meets the needs of all patients. We must be highly specialized and rigorous in both what we do and how we do it, maintaining focus during both stable periods and crises.

- 1. Standardizing Clinical Processes

To ensure a consistent approach, we will focus on standardizing clinical processes to reduce variability. This involves:

- **Developing Clear Protocols:** Establishing explicit protocols and implementing necessary alerts.
- **Investing in Data:** Prioritizing continuous, accurate data collection for comprehensive analysis, diagnosis, and action planning. We must clearly define our current status, objectives, and methods, guided by detailed plans.
  1. Building a Strong Foundation

Our strategy includes following a structured and methodical process, supported by robust planning and the use of top-quality materials. Key elements include:

- **Assembling a Skilled Team:** Bringing together a team of highly qualified professionals.
- **Supervision by Experts:** Ensuring oversight by an experienced allergist specializing in the technical area.
- **Focusing on Risk Management:** Emphasizing strong risk management, patient safety, and staff protection, with a particular focus on anaphylaxis prevention.
- **Preparing for Complex Cases:** Being ready to handle complex diseases, severe conditions, and ongoing service improvements.
  1. Implementing Operational Standards

To ensure consistent and high-quality care, we will establish clear operational standards that must be followed by all staff. This includes:

- **Daily Checklists:** Utilizing checklists to ensure completion of all essential tasks.
- **Special Procedure Records:** Keeping detailed records of all special procedures.
- **Clinical Documentation:** Ensuring comprehensive clinical documentation, including informed consent forms.
- **Clear Protocols:** Developing straightforward, practical, written protocols to reduce variability in care. These will cover:
  - Clinical pathways
  - Clinical guidelines
  - Organizational procedures
  - Procedure manuals

Standardization will not only involve creating these protocols but also promoting the right attitudes and practices among staff to ensure they are consistently applied.

- 1. Designing Patient Management Systems

To enhance the efficiency of our allergy division, we must clearly define and design patient management algorithms, protocols, and flowcharts.

These should be applied to individual cases, and every patient should be booked with a request form that tackles the following key aspects:

- **Clear Diagnostic Requests:** Ensure that patients arrive at the technical area with a specific diagnostic or therapeutic request.
- **Detailed Risk Stratification:** Each request should be accompanied by a thorough risk assessment conducted by the prescribing physician.
- **Designated Area Assignment:** Assigning patients to appropriate areas (red, yellow, green, or premium) based on their risk levels.

By implementing these measures, we can streamline patient flow within the allergy division, ensuring that each patient receives appropriate care based on their risk level.

This way, patients should arrive at the technical area with a clear diagnostic or therapeutic request accompanied by a completed risk stratification. This information will determine the appropriate placement of the patient within the technical area (e.g., Red, Green, Yellow, or Premium room) for the duration of the planned procedure.

**TABLE 1: Risk Assessment Matrix for the Pediatric Allergy Technical Area for Diagnostic and Therapeutic Procedures**

| **Criteria** | **High Risk (Red)** | **Moderate Risk (Yellow)** | **Low Risk (Green)** | **Minimal Risk (White)** |
| --- | --- | --- | --- | --- |
| **Severity of Allergies** | Severe reactions | Moderate reactions | Mild reactions | No significant allergies |
| **Comorbidities** | Severe comorbidities | Moderate and well-controlled comorbidities | Minor and well-controlled comorbidities | No comorbidities |
| **Complexity of Procedure** | High complexity, advanced techniques, complex preparations (e.g. complex skin testing or drugs) | Moderate complexity, standard techniques | Low complexity, minimal risks | Very simple procedure, negligible risks |
| **Patient Age and Condition** | Severe conditions, very young, acutely ill, or frail patients | Moderate conditions, average age and health | Mild conditions, stable health | Very mild conditions, stable health |
| **Procedure Type** | High-risk drug or food challenges or desensitizations | Routine procedures with moderate risk | Low-risk diagnostic or therapeutic procedures | Very basic and low-risk procedures |
| **Room Type** | Premium Rooms (beds), Red Rooms (recliners with CPR mode and CPR stretcher) | Yellow Rooms (recliners and CPR stretcher) | Green Rooms (comfortable chairs and waiting area) | Minimal Risk Rooms (if available), or green rooms |
| **Supervision Required** | Highly specialized supervision, one nurse per 1-2 patients, allergist available at all times | Regular supervision, one nurse per 3-5 patients, allergist available | Standard supervision, one nurse per 6-10 patients, less frequent monitoring | Minimal supervision, general monitoring |
| **Equipment Needs** | Vital signs monitors, central monitoring station, resuscitation cart, defibrillator, advanced treatment equipment | Vital signs monitors, basic treatment equipment | Basic monitoring equipment | Minimal equipment needed |
| **Precautions** | Continuous monitoring, ready access to emergency care, advanced anaphylaxis protocols | Regular monitoring, basic emergency protocols | Standard safety measures | Minimal safety measures |
| **Room Safety** | High safety standards, advanced emergency protocols | Standard safety measures, emergency protocols | Basic safety measures | Basic safety measures |
| **Room Assignment** | Premium Rooms for high-dependency care, Red Rooms for high-risk procedures | Yellow Rooms for moderate risk | Green Rooms for routine care | Minimal Risk Rooms if needed |
| Note: Only for guidance—local variation is expected depending on spaces, staffing, and resources | | | | |

- 1. Fostering a Culture of Excellence in the Allergy Division

We must never underestimate what many consider the "mother of all battles"—the cultural battle (1-2). To achieve true excellence in our Allergy Division, we will cultivate a culture rooted in safety, particularly the prevention of anaphylaxis, alongside an unwavering commitment to quality and continuous improvement. This culture will emphasize:

- Knowledge-Sharing
- Teamwork
- Dedication
- Collaborative Decision-Making

Conversely, we will reject a culture of minimal effort, which leads to mediocrity. This mindset, seeking rewards without the necessary effort, undermines passion and excellence. To combat mediocrity, we must create an environment driven by passion and clear, measurable, purposeful goals.

The guiding workplace ethos of our Allergy Division aligns with the Japanese concepts of "Ganbaru" and "Kaizen." "Ganbaru" involves perseverance, effort, and determination, capturing the essence of doing one's best and persisting through challenges. We modernize this with elements of "Kaizen"—continuous improvement—and emphasize working with passion, commitment, and clear objectives. Additionally, we advocate for a balanced approach, ensuring efficiency at work so as to allow for an appropriate work-life balance that includes time for personal well-being, recognizing the importance of returning to family and disconnecting after work.

By "thinking globally and acting locally," we aim to foster a win-win relationship with our medical and nursing staff, ensuring that our collective actions align with our goals of providing exceptional patient care and achieving operational excellence.

# Performance:

- 1. Commitment to Efficiency

Our commitment is to efficiency, as it guarantees our present, our current success and future stability. We consistently focus on achieving results, with a clear emphasis on premium objectives and resolving issues effectively.

- 1. Establishing and Monitoring Objectives

To ensure that we meet the defined objectives, it is crucial to establish ratios and indicators, monitor them, assess and analyze the data, and take action based on the proposed goals. As William Thomson Kelvin, the 19th-century British physicist and mathematician, famously stated: "What is not defined, can not be measured. What is not measured can not be improved. What is not improved, will always degrade".

- 1. Standards-Based Framework and Goal Orientation

Evidence shows that the highest performance is achieved when operating under a "standards-based" framework and goal-oriented approach. Goals should be systematically evaluated using indicators, followed by an analysis of results from this technical area, and these results should be made public (29). It is important to view monitoring and evaluation not as corrective tools, but as valuable resources that support our continuous improvement efforts.

- 1. Selecting and Using Quality Indicators

Regarding indicators, such as patient satisfaction, safety, efficiency, and staff-to-patient ratios, we should select a set of approximately 30 evidence-based and expert-supported indicators for evaluating care quality in the Allergy technical area. These indicators should cover diagnosis, treatment, risk management, and health outcomes.

- 1. Additional Considerations

Other critical aspects to consider include:

- Administrative Efficiency: Is bureaucracy being managed efficiently?
- Role of Administrative Staff: Is the role of administrative personnel being maximized?
- Reduction of Bureaucracy: Can we reduce bureaucratic processes?
- Digital Health: How is the digital health of the Pediatric Allergy Division/Technical Area?
- Information Systems: Are information systems used effectively for data exchange?
- Win-Win Principle: Is the "Win-Win" principle upheld in the Pediatric Allergy Division/Technical Area? In other words, do we prioritize the team's success over individual achievements?

By addressing these aspects, we can ensure comprehensive performance management and continuous enhancement of the Allergy Division.

# Price - Financial Planning and Management

- 1. Cost-Effectiveness in Pediatric Allergy and Clinical Immunology

Our extensive experience confirms that with effective management of the Pediatric Allergy and Clinical Immunology Technical Area, cost-effectiveness won't be a problem. However, it is crucial to remember the principle of Henry Ford: "A business that makes nothing but money is a poor business." This highlights the importance of balancing financial goals with the overall quality and purpose of the allergy division.

- 1. Developing a Comprehensive Business Plan

A well-structured, written, and regularly evaluated business plan is essential. Key components of this plan should include:

- **Project Vision and Implementation Plan:** Clearly outline the project's vision and create a plan to bring it to fruition.
- **Current State Analysis:** Assess the current status of the business, including the general momentum, desired outcomes, and unique value propositions at that moment.
- **Setting Clear Objectives:** Define specific, actionable goals, prioritize them, and regularly evaluate progress towards these objectives.
- **Decision-Making Flexibility:** Make decisions that adapt to evolving circumstances and ensure financial planning is adaptable.
- **Financial Plan:** Develop a detailed financial plan covering weekly, monthly, and annual expenses.

Effective financial planning and management are critical to ensuring the success and sustainability of the Pediatric Allergy and Clinical Immunology Division. By adhering to these principles and balancing financial considerations with the core values of the allergy division, we can achieve both operational efficiency and quality care.

# Perspecti**ve - Focus on Growth Drivers**

- 1. Embracing Growth as a Catalyst

Commitment to growth is a proactive step towards fostering positive change. Our efforts will center on driving advancements through several key areas: skill development, continuing medical education, digital transformation, continuous improvement, technological innovation, and research.

- 1. Skill Development and Professional Training

Investing in skill development and professional training is vital for maximizing human potential. Continuous learning and skill enhancement contribute to greater efficiency and creativity. Tools such as artificial intelligence, automation, and collaborative software enable faster and more accurate task completion. When integrating new technological tools or systems, preparing users through training is essential. Technology's effectiveness depends on the proficiency of its users, necessitating investments in comprehensive training sessions to ensure familiarity and comfort with new tools.

- 1. Digital Transformation

A strategy focused on digitalization has revolutionized operational processes, enhancing efficiency, streamlining workflows, optimizing time management, and reducing costs. Effective use of technology is crucial, and its integration into technical areas, such as embedding protocols into electronic health records, will facilitate data utilization for research and management.

- 1. Technological Innovation

Investing in technological innovation is crucial for ensuring future success. It is unacceptable to halt improvements or innovations due to reluctance to consider feedback from those who observe processes, analyze data, and understand technical concepts.

Leaders must allocate time to meet with colleagues to understand their insights and experiences. Pragmatism dictates starting with simple solutions and scaling up, ensuring that innovative individuals are not frustrated by delays or lack of priority.

Innovation thrives in a culture that supports experimentation and learning from failures, distinguishing between good and transformative ideas that align with patient needs, company strategy, and economic sense.

- 1. Ongoing Medical Education

The continuing education of medical and nursing staff will be centered on the "case method" from Harvard University and technical proficiency. The goal is not merely to pass exams but to create, complete, and publish projects (project-based learning and evaluation). Patient education, including preventive care, will also be emphasized.

- 1. Continuous Improvement

Continuous improvement involves systematically enhancing products, services, and operations through standardized practices. This approach ensures that the allergy division and the technical area remain aligned with the established objectives and priorities set by management. Continuous improvement helps overcome challenges and meet expectations.

- 1. Medical Research

Medical research seeks rational, endless solutions to health problems. It is imperative to persist in finding answers, as failure to do so can result in stagnation. "Between an impossible and a possible, there is research."

- 1. Establishing a Clinical Research Unit

We will establish a Clinical Research Unit within the Allergy Division (CRUAD) to support researchers in developing projects. This unit will provide researchers with the necessary facilities and resources to perform their work effectively and safely. The unit will focus on the researcher's needs, with funding sourced from research projects. Upon arrival, the researcher will find the project's data collection notebook on the table, with the patient/volunteer already present. The researcher will conduct their work without administrative or nursing tasks, which should be completed beforehand. Local needs and priorities will be defined and addressed accordingly (31).

# Projects - The Cornerstones for Problem Solving

- 1. Defining Projects versus Processes

Projects are fundamental for addressing the challenges we face. It is important to differentiate between a process and a project. A project is unique, with specific requirements and a defined timeframe for achieving its intended outcome. In contrast, a process is ongoing and does not have a fixed end date.

- 1. Project Framework

When a question or idea arises, a project is constructed to address the core issue. The key is to have a clear understanding of what needs to be done and how to implement it. Projects are concrete objectives based on irrefutable data, with a structured and rigorous plan and unavoidable goals. They must be realistic and achievable.

- 1. Purpose and Types of Projects

Projects serve as our primary tools for motivation, progress, and problem resolution, making them the cornerstones of our environment. They can be structural/strategic, operational, supportive, translational research, or educational.

- 1. Project Management and Execution

Projects must be result-oriented, with the ultimate goal of solving the problem at hand. To achieve this, we need to:

1. **Define the Problem:** Clearly outline and characterize the problem.
2. **Develop a Solution:** Propose and quantify a solution.
3. **Create a Timeline:** Develop a detailed schedule.
4. **Implementation Plan:** Document how the project will be executed.
5. **Goal Achievement:** Establish methods for measuring and meeting the project goals.

# PubMed - The Importance of Publication

**Leadership in research publications should be a fundamental priority.** Research and experience that are not published are, in effect, non-existent. It is crucial to understand that unpublished research and experiences, no matter how significant, will not contribute to the scientific community or the advancement of knowledge.

- 1. Maximizing Publication Impact

It is imperative to adopt a proactive approach to publishing. This involves:

- **Prioritizing Publication:** Ensure that all significant research findings, audits, and experiences are published. This is not just a matter of personal or institutional prestige but a critical component of academic and professional growth.
- **Strategic Planning:** Develop a strategic plan for disseminating research results. This should include selecting appropriate journals, understanding submission requirements, and preparing high-quality manuscripts.
- **Engagement with the Scientific Community:** Actively participate in conferences, workshops, and seminars. These platforms not only provide opportunities for presenting research but also for networking and collaborating with peers.
- **Regular Reviews:** Continuously review and update publication strategies to align with the latest research trends and requirements. This helps maintain relevance and ensures that research findings reach the intended audience effectively.

# Summary of Key Recommendations for Designing and Optimizing a Pediatric Allergy Technical Area

**1. Embrace the Experience of Creating an Exemplary Pediatric Allergy Division**

We invite the reader to enjoy and fully engage in the life experience that is the process of creating, redesigning, or refining a pediatric diagnostic and therapeutic technical area. This endeavor represents a paradigm shift for both the patient and the allergist. From the authors' perspective, undertaking this practice has been immensely motivating, rewarding, and an experience worth undertaking.

**2. Conduct a Thorough Review Before Redesign**

Before embarking on any redesign or optimization of the technical area, it is crucial to conduct a comprehensive review of current operations. This involves obtaining and analyzing reliable and accurate information, periodically assessing and updating key elements discussed in this document, making an accurate diagnosis of the current situation, and establishing a clear roadmap. Such preparation will enable effective planning and execution of the redesign process.

**3. Define the Operational Framework**

The co-leaders of the Allergy and Clinical Immunology specialty (the technical area lead and the allergy division lead) must clarify the operational framework for handling the rapidly increasing referrals for allergic conditions. They should determine whether the focus will be on managing complex, severe, high-risk patients or if these cases should be referred to a tertiary referral hospital. It is essential to outline a clear roadmap, define key points of focus, and establish operational rules.

**4. Prioritize the Development of a Well-Equipped and Well-Staffed Allergy Technical Area**

A well-equipped, well-designed, and adequately staffed Allergy Technical Area is fundamental to the success of the specialty. It is essential to periodically transform and enhance the technical area with a focus on the following core principles: "safety first," anaphylaxis prevention, and ensuring both high-quality and compassionate patient care.

**5. Commit to Continuous Improvement**

Continuous improvement requires ongoing work that includes reflection and consistent self-evaluation. This commitment to improvement is essential for maintaining and enhancing the effectiveness and efficiency of the allergy division.

**6. Balance Efficiency and Innovation**

Both efficiency and innovation are crucial considerations. Efficiency ensures the sustainability of current operations, while innovation guarantees future progress and adaptation to emerging needs and challenges.

**7. Establish and Adhere to Excellent Rules and Principles**

It is vital to establish clear rules and a set of principles to guide operations. Additionally, transparency and a clear monitoring system for tracking the progress of commitments are necessary for maintaining accountability and ensuring continuous improvement.

**TABLE 2: Quality Indicators that can be collected to create a departmental Quality Assurance Metrics Dashboard**

| Category | Indicator | Description | Type of Chart |
| --- | --- | --- | --- |
| **Patient Satisfaction** | Overall Patient Satisfaction Score | General satisfaction with the services received. | Bar Chart / Line Graph |
|  | Satisfaction with Waiting Times | Feedback on wait times for appointments and procedures. | Bar Chart / Pie Chart |
|  | Satisfaction with Communication | Satisfaction with how well patients are informed and communicated with. | Bar Chart / Line Graph |
|  | Satisfaction with Facility Comfort | Ratings on the comfort and cleanliness of the facility. | Bar Chart / Pie Chart |
| **Safety** | Incidence of Adverse Events | Frequency of adverse reactions or incidents during procedures. | Line Graph / Bar Chart |
|  | Anaphylaxis Management Success Rate | Effectiveness of managing anaphylactic reactions. | Line Graph / Bar Chart |
|  | Compliance with Safety Protocols | Adherence to established safety procedures and protocols. | Bar Chart / Compliance Dashboard |
|  | Rate of Medication Errors | Frequency of errors in medication administration. | Bar Chart / Line Graph |
|  | Infection Rate | Incidence of infections related to procedures or hospital stays. | Line Graph / Bar Chart |
|  | Rate of Safety Alerts Issued | Frequency of safety alerts or notifications issued. | Bar Chart / Line Graph |
| **Efficiency** | Average Wait Time for Appointments | Average time patients wait for an appointment. | Line Graph / Bar Chart |
|  | Average Procedure Time | Average duration of allergy-related procedures. | Line Graph / Bar Chart |
|  | Staff-to-Patient Ratio | Ratio of staff members to patients. | Bar Chart / Line Graph |
|  | Resource Utilization | Efficiency in using medical and facility resources. | Bar Chart / Pie Chart |
|  | Appointment No-Show Rate | Percentage of missed appointments. | Line Graph / Bar Chart |
|  | Bed Occupancy Rate | Percentage of available beds occupied in the technical area. | Line Graph / Bar Chart |
| **Quality of Care** | Rate of Correct Diagnoses | Percentage of accurate diagnoses. | Bar Chart / Pie Chart |
|  | Rate of Successful Treatments | Success rate of treatments administered. | Bar Chart / Line Graph |
|  | Protocol Adherence Rate | Compliance with clinical protocols and guidelines. | Compliance Dashboard / Bar Chart |
|  | Rate of Follow-Up Care | Percentage of patients receiving appropriate follow-up care. | Bar Chart / Line Graph |
|  | Patient Education Effectiveness | Effectiveness of educational materials and sessions provided. | Bar Chart / Pie Chart |
|  | Success Rate of High-Risk Procedures | Success rate of procedures classified as high-risk. | Bar Chart / Line Graph |
| **Risk Management** | Risk Assessment Accuracy | Precision of risk assessments prior to procedures. | Bar Chart / Line Graph |
|  | Emergency Response Time | Average time taken to respond to medical emergencies. | Line Graph / Bar Chart |
|  | Incident Reporting Rate | Frequency of reported incidents or near-misses. | Line Graph / Bar Chart |
|  | Quality of Risk Communication | Effectiveness of communicating risks to patients and families. | Bar Chart / Line Graph |
| **Outcome** | Clinical Outcome Measures | Measures of clinical outcomes such as symptom improvement. | Line Graph / Bar Chart |
|  | Rate of Complications | Frequency of complications from procedures or treatments. | Bar Chart / Line Graph |
|  | Patient Recovery Time | Average time for patients to recover from procedures. | Line Graph / Bar Chart |
|  | Readmission Rate | Percentage of patients readmitted within a specified period. | Line Graph / Bar Chart |
| **Operational** | Operational Efficiency Score | Evaluation of operational efficiency in the Allergy Technical Area. | Line Graph / Bar Chart |
|  | Staff Training Compliance | Percentage of staff completing required training and certifications. | Bar Chart / Line Graph |
|  | Patient Flow Efficiency | Effectiveness of patient flow through the technical area. | Flow Chart / Bar Chart |
|  | Average Length of Stay | Average duration of patient stay in the technical area. | Line Graph / Bar Chart |
| **Patient Demographics** | Patient Age Distribution | Distribution of patients by age group. | Pie Chart / Bar Chart |
| **Patient Experience** | Net Promoter Score (NPS) | Measure of patient loyalty and likelihood to recommend the service. | Bar Chart / Line Graph |
| **Financial Metrics** | Cost per Procedure | Average cost incurred for each procedure performed. | Bar Chart / Line Graph |
| **Service Utilization** | Procedure Utilization Rate | Frequency of different procedures performed relative to their availability. | Bar Chart / Pie Chart |
| **Clinical Research** | Number of Clinical Trials Conducted | Total number of clinical trials or studies performed. | Bar Chart / Line Graph |
| **Technology Utilization** | Rate of Technology Adoption | Rate at which new technologies or systems are adopted and used. | Line Graph / Bar Chart |
| **Staff Well-Being** | Staff Turnover Rate | Rate at which staff leave the department. | Line Graph / Bar Chart |
| **Patient Access** | Time to First Appointment | Average time from initial contact to the first appointment. | Line Graph / Bar Chart |
| **Managerial** | Number of audits and quality improvement (QI)projects per year | It measures the number of QI or research projects that have studied activitiy in the technical area | Line Graph / Bar Chart |
|  | Annual activity and efficiency report at departmental clinical governance meeting | It confirms whether data are openly shared and discussed with all the department every year | Line Graph / Bar Chart |
|  | Number of annual operational meetings between multidisciplinary team leads and managers | It confirms a continuous engagement to monitor performance | Line Graph / Bar Chart |

**References:**

1. Alvarez-Cuesta E., Berges-Gimeno M, Cuesta-Herranz J. Área de procedimientos alergológicos, diagnósticos y terapéuticos en el siglo XXI: propuesta de un modelo estándar. In: Guzman-Melendez A. ed. Alergia e Intolerancia Alimentaria. Santiago de Chile: Editorial Mediterráneo: 2015:413-423. https://mediterraneo.cl/busqueda?controller=search&order=product.position.desc&s=978-956-220-371-5. Accessed February 11, 2021.
2. Alvarez-Cuesta E. Madrigal-Burgaleta R. Technical area for diagnostic and therapeutic procedures in an allergy department. World Allergy Organization Journal (2022) 15:100640.- Supplementary text 1, 1-18. http//doi.org/10.1016/J.WAOJOU.2022.100640
3. WHO/IUIS Allergen Nomenclature Sub-Committee. Allergen nomenclature. www.allergen.org 2023.
4. 4Jutel M., Agache I. Zemelka-Wiacek M. et al. Nomenclature of allergic diseases and hypersensitivity reactions: adapted to modern needs. Allergy 2023;00:1-24 Doi: 10.1111/all.15889
5. Mount Sinai Expert Guides: Allergy and Clinical Immunology, first edition. Edited by Hugh A. Sampson. 2015 John Wiley & Sons, Ltd. Published 2015 by John Wiley & Sons, Ltd. Companion webside: [www.mountsinaiexpertguides.com](http://www.mountsinaiexpertguides.com)
6. Sawyer T, McBride M.E., Ades A. Considerations on the use of neonatal and pediatric resuscitation guidelines for hospitalized neonates and infants: on behalf of the American Heart Association Emergency Cardiovascular Care Committee and the American Academy of Pediatrics. Pediatrics 2024, 153, 67-81:e2023064681
7. Kowalski ML. Ansotegui I. Aberer W. et al. Risk and safety requirements for diagnostic and therapeutic procedures in allergology: World Allergy Organization Statement. World Allergy Organ J. 2016;9(1):1-42. doi:10.1186/s40413-016-0122-3.
8. Cardona V, Ansotegui IJ, Ebisawa M et al. World allergy organization anaphylaxis guidance 2020. World Allergy Organ J. 2020;13(10):100472.doi:10.1016/j.waojou.2020.100472.
9. Shaker MS, Wallace DV., Golden DBK et al. Anaphylaxis -a 2020 practice parameter update, systemic review, and Grading of recommendations, Assessment, Development and Evaluation (GRADE) analysis. J. Allergy Clin. Immunol. 2020;145(4):1082-1123. Doi:10.1016/j.jaci.2020.01.017
10. Alvarez-Cuesta E. R.Madrigal-Burgaleta et al. Standards for practical intravenous rapid drug desensitization & delabeling: A WAO committee statement. World Allergy Organization Journal (2022) 15:100640.- 1-66. http//doi.org/10.1016/ J.WAOJOU.2022.100640
11. Ricardo Madrigal….[Alvarez-Cuesta E.]. "A large single-hospital experience using drug provocation testing and rapid drug desensitization in hypersensitivity to antineoplastic and biological agents". J Allergy Clin Immunol Pract 2019; 7: 618-632. Doi:10.1016/j.jaip.2018.07.031
12. David I Hong... [Alvarez-Cuesta E.] Controversies in Allergy: chemotherapy reactions, desensitize or delabel. J. Allergy Clin Immunol Pract 2020, 8: 2907-2915; doi.org/10.1016/j.jaip.2020.08.005;
13. Anca M. Chiriac, MD, PhD, Aleena Banerji, MD, Rebecca S. Gruchalla, MD, PhD, Bernard Y.H. Thong, MBBS, MRCP, FRCP, Paige Wickner, MD, MPH, Paul-Michel Mertes, MD, PhD, Ingrid Terreehorst, MD, PhD, and Kimberly G. Blumenthal, MD, Controversies in Drug Allergy: Drug Allergy Pathways. J Allergy Clin Immunol Pract. 2019; 7:46-60.
14. Sampson HA, Aceves S, Bock et al. Food allergy: a practice parameter update -2014. J. Allergy Clin Immunol 2014;134:1015-25
15. Canonica AF, et al. EAACI guidelines on the diagnosis of IgE-mediated food allergy. Allergy. 2023;00:1-20. doi:10.1111/all.15902.
16. Sturm G.J., Varga E-M, Roberts G et al. EAACI guidelines on allergen immunotherapy: hymenoptera venom allergy. Allergy 2018: 73:744-764.
17. Golden D., Moffitt J., Nicklas R. et al Stinging insect hypersensitivity: a practice parameter update 2011. J. Allergy Clin Immunol 2011 Apr;(4):852-4.e 1-23 doi 10.1016/j.jaci.2011.01.025 (http:www.aaaai.org/conditions-and- treatments/allergies/stinging-insects-allergy.aspx)
18. Ruëff F, et al. Diagnosis and treatment of Hymenoptera venom allergy. Allergol Select. 2023; 7: 154-190. DOI 10.5414/ALX02430E
19. Alvaro-Lozano M, Akdis C.A., Akdis M. et al EAACI allergen immunotherapy user's guide Pediatr Allergy Immunol off Publ Eur Soc Pediatr Allergy Immunol 2020; 31:1-101
20. James Christine and Bernstein David I. Allergen Immunotherapy: an updated review of safety. Curr Opin Allergy Clinical Immunol 2017 Feb; 17(1):55-59. Doi:10.1097/ACI.0000000000000335
21. Cox L, Nelson H, Lockey R et alAllergen immunotherapy: a practice parameter third update J Allerg Clin Immunol 2011;127 (1 Suppl): S1-S55. (http:www.aaaai.org/Aaaai/media/MediaLibrary/PDF%20Documents/Practice%20and%20Parameters/Allergen-immunotherapy-Jan-2011.pdf)
22. Solano Solares E… [Alvarez-Cuesta E.] "Chemotherapy in Mastocytosis: Administration Issues, Hipersensitivity, and Rapid Drug Desensitization". J Investig Allergol Clin Immunol 2017; 27 (5): 315-317. Doi.10.18176/jiaci.0171
23. Alvarez-Cuesta E., Madrigal-Burgaleta R. et al. "Delving into a cornerstones of hypersensitivity to antineoplastic and biological agents: value of diagnostic tools prior to desensitization". Allergy 2015; 70: 784-794. Doi:10.1111/all.12620
24. Alvarez-Cuesta E., J. Bousquet, GW Canonica, SR Durham, HJ Malling, E. Valovirta. "Standars for practical allergen-specificimmunotherapy". EAACI Immunotherapy Task Force. Allergy Eur J. Allergy Clin. Immunol 2006; 61 (Suppl 82): 1-20. Doi:10.1111/j.1398-9995.2007.01302.x
25. Alvarez Cuesta E., P. Berges Gimeno, EG Mancebo, E. Fernandez Caldas, J. Cuesta Herranz, M. Casanovas. "Sublingual immunotherapy with a standarized cat dander extract: evaluation of efficacy in a double blind placebo controlled study". Allergy 2007: 62: 810-817
26. Alvarez Cuesta E, J. Cuesta Herranz, J. Puyana Ruiz, C. Cuesta Herranz, A. Blanco Quiros. "Monoclonal antibody-standarized cat extract immunotherapy: Risk-benefit effects from a double-blind placebo study". J Allergy Clin Immunol 1994; 93: 566-66
27. Bousquet J, Lockey R.F.,Mallin H-J et al (Integra Panel Members). WHO Position paper "Allergen Immunotherapy: Therapeutic vaccines for allergic diseases" Allergy 1998; 53 (Suppl): 1-42
28. Alvarez-Cuesta E..Madrigal-Burgaleta R. Berges-Gimeno M.P. Angel-Pereira D. Rapid desensitization to chemotherapy and monoclonal antibodies is effective and safe. Reply. Allergy. 2013; 68: 1482-1484. doi:10.1111/all.12228
29. Bergbauer A.B., Hanushek EA, Woessmann L., “Testing, Journal of Human Resources, 2024, pages 349-388.
30. Cruz Hernandez JJ, Arrazubi Arrula V., Escobar Alvarez Y., et al. Indicators to evaluate quality of care in head and neck cancer in Spain. Clinical and Translational Oncology (2023). https:doi.org/10.1007/s12094-023-03298-z
31. Papadopoulos NG, Agache I, Bilo BM et al Research needs in Allergy: an EAACI position paper, in collaboration with EFA.- Clinical and translational Allergy 2012, 2:21 DOI: 10.1186/2045-7022.2-21
