# Supplementary material for: Drug hypersensitivity reactions in children in clinical practice: A WAO Statement
Source: World Allergy Organ J. 2025 Aug 29;18(9):101087. doi: 10.1016/j.waojou.2025.101087 (PMC12419032; doi:10.1016/j.waojou.2025.101087)
Supplement: Multimedia component 3 [file mmc3.docx]

**SUPPLEMENTAL APPENDIX 2: Evaluation and management of antiepilectic DHR in pediatric populations**

HIGHLIGHTS

1. Antiepileptic drugs(AEDs) are the mainstay of epilepy therapy but their use in non-epilepsy disorders such as psychiatric illnesses, addiction and pain disorders is rapidly increasing, especially among adolescents. Sodium valproate, carbamazepine and lamotrigine are the most commonly employed agents for pediatric epilepsy. However newer agents, especially levetiracetam, are on the rise.
2. Drug metabolism and interactions are important for AEDs and genetic and non-genetic metabolic derangements may predispose to every type of adverse reactions. Drug interactions are lower for new generation drugs
3. The incidence and prevalence of hypersensitivity reactions(HSR) to AEDs in children is mostly unknown. However, it was recently shown that 1 to 6 per 100 children started on therapy has developed HSRs, of which nearly 20% was severe, in a limited number of prospective real-life studies in UK and Turkey
4. Preschool-early school age, aromatic structure, a high starting dose, rapid dose escalation and multi-AED regimens significantly increases the risk of AED-HSRs.
5. Limited number of pediatric studies showed associations between HLA-B*13:01 and phenobarbital hypersensitivity in Thai children; HLA-B*15:02 and CBZ-induced SCAR in children of Chinese and Malay ethnicity; HLA-A*31:01 and CBZ-induced DRESS and MPE in European and North American children.
6. Every type of HSR can be expected but majority are mild non-immediate skin reactions in the form of maculopapular rash and urticaria
7. AEDs, especially aromatic ones, are among the top three agents related to severe cutaneous adverse drug reactions(SCARs) and drug-induced fatality in children . Organ-specific reactions should not be overlooked
8. An endophenotypic diagnostic algorithm including in vivo prick and intradermal tests, patch tests and provocation tests and in vitro tests, if available, should be employed to confirm or exclude AED-HSR (Figure 2)
9. Drug patch tests are safe and have a relatively high diagnostic value to identify the suspected AED in delayed reactions
10. In SCARs patch tests are the first line of diagnostic algorithm and a relatively safe test in children. Delayed reading of intradermal tests can be employed in AGEP and rare selected cases of DRESS, but not in SJS/TEN and other severe reactions. Drug provocation test is contrindicated in all severe reactions including SCARs
11. Cross reactivity between aromatic AEDs is high and aromatics should be avoided in confirmed or suspected cases of HSR to another aromatic AED

GAPS IN KNOWLEDGE

1. Studies on incidence, prevalance and risk factors for AED-HSR in children are still lacking in most of the populations
2. The effect of metabolic differences specific to children on incidence of hypersensitivity to AEDs is not known
3. Worldwide HLA studies on AED-HSR among children are lacking
4. Standardized non irritant maximum prick and intradermal tests and, to some extent, patch test concentrations for AEDs are unknown
5. Optimal AED provocation doses and intervals are unknown
6. Prognosis of AED-HSRs in children is unknown
7. There is a critical need for large-scale studies in children with standardized methodology to obtain informative data about epidemiology, risk factors, diagnosis and prognosis of AED-HSR in children
